# Analyzing the Quality Parameters of Apples by Spectroscopy from Vis/NIR to NIR Region: A Comprehensive Review

**DOI:** 10.3390/foods12101946

**Published:** 2023-05-10

**Authors:** Justyna Grabska, Krzysztof B. Beć, Nami Ueno, Christian W. Huck

**Affiliations:** Institute of Analytical Chemistry and Radiochemistry, University of Innsbruck, Innrain 80-82, 6020 Innsbruck, Austria; justyna.grabska@uibk.ac.at (J.G.); christian.w.huck@uibk.ac.at (C.W.H.)

**Keywords:** apple, quality control, Vis/NIR spectroscopy, NIR spectroscopy, portable/handheld spectrometers, external and internal quality parameters

## Abstract

Spectroscopic methods deliver a valuable non-destructive analytical tool that provides simultaneous qualitative and quantitative characterization of various samples. Apples belong to the world’s most consumed crops and with the current challenges of climate change and human impacts on the environment, maintaining high-quality apple production has become critical. This review comprehensively analyzes the application of spectroscopy in near-infrared (NIR) and visible (Vis) regions, which not only show particular potential in evaluating the quality parameters of apples but also in optimizing their production and supply routines. This includes the assessment of the external and internal characteristics such as color, size, shape, surface defects, soluble solids content (SSC), total titratable acidity (TA), firmness, starch pattern index (SPI), total dry matter concentration (DM), and nutritional value. The review also summarizes various techniques and approaches used in Vis/NIR studies of apples, such as authenticity, origin, identification, adulteration, and quality control. Optical sensors and associated methods offer a wide suite of solutions readily addressing the main needs of the industry in practical routines as well, e.g., efficient sorting and grading of apples based on sweetness and other quality parameters, facilitating quality control throughout the production and supply chain. This review also evaluates ongoing development trends in the application of handheld and portable instruments operating in the Vis/NIR and NIR spectral regions for apple quality control. The use of these technologies can enhance apple crop quality, maintain competitiveness, and meet the demands of consumers, making them a crucial topic in the apple industry. The focal point of this review is placed on the literature published in the last five years, with the exceptions of seminal works that have played a critical role in shaping the field or representative studies that highlight the progress made in specific areas.

## 1. Introduction

**Figure 1:**
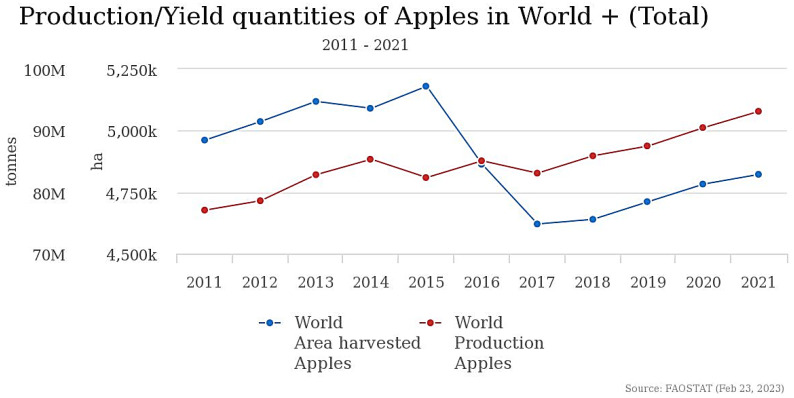
Apple production quantities worldwide from 2010 to 2021. Reprinted with permission from Ref. [1]. 2023, World Population Review.

Apples are one of the most widely produced and consumed crops in the world (Figure 1), with China being the largest producer followed by the United States and Poland [1]. In recent years, there has been a growing interest in the nutritional value of apples and their health benefits, which has driven up demand for this fruit. Furthermore, advances in storage techniques, such as the use of 1-methylcyclopropene (1-MCP) [2], have allowed apples to be stored for months at low temperatures, thereby extending their shelf life and increasing their availability throughout the year. However, despite these advances, the apple industry continues to face challenges due to climate change and the need for quantitative control of production.

The changing climate and local weather conditions have made it increasingly difficult for apple orchards to maintain consistent production levels [3]. As a result, farmers are forced to adapt to these changing conditions by implementing new controlled methods that take into account various parameters, such as temperature, sunlight, and chlorophyll concentration. This quantitative control not only improves the quality of the apples but also helps farmers to reduce waste by throwing away bad or low-quality apples early on. In addition, the use of optical methods, such as spectroscopy and computer vision, can further aid in the detection of diseases and other quality issues during storage and transportation.

To maintain their profits, apple farmers must employ various strategies, including producing high-quality apples, reducing waste, and reducing production costs. Optical methods are a powerful analytical tool that can support all of these strategies. For example, optical sensors can be used to sort fruits by the quality and detect any diseases early on, enabling farmers to pick off infected fruits and protect the remaining ones. Additionally, the use of handheld instruments and smartphone applications can make these methods more accessible to farmers and consumers alike [4]. Finally, given the increasing competition within the apple industry, it is crucial to sell high-quality apples at a low price, which can be achieved through the use of optical methods to improve grading and sorting processes.

To meet the requirements of the apple industry, an analytical technique should possess a set of advantageous characteristics [5,6,7,8,9,10]. The analytical technique should be able to support the quantitative control of apple production and aid in the detection of diseases and other quality issues, helping farmers to produce high-quality apples, reduce waste, and sell their products at a competitive price. Most of those requirements can be satisfied by applying optical sensors operating in near-infrared (NIR) and Visible/NIR (i.e., Vis/NIR) wavelength regions [5,6,7,8,9,10].

NIR spectroscopy has been widely used to analyze various food products, including fruits such as apples, for their quality evaluation and quantification. In apple analysis, NIR spectroscopy has been used to measure various quality parameters [11,12,13,14,15]. This technique has the potential to provide rapid, accurate, and non-destructive analysis of apples and could be used in quality control and sorting operations in the fruit industry [16,17,18,19]. The first report on NIR in postharvest biology was by the founder of the NIR discipline, Karl Norris who demonstrated the use for apple internal rot detection in the early 1960s [20]. NIR spectroscopy is considered one of the most potent analytical techniques and is widely used as a non-destructive method in analyzing various properties of raw materials and products in several fields [21]. Often NIR spectroscopy is combined with the chemometrics method to evaluate the quality of the food [22]. Several studies have shown the potential application of NIR spectroscopy to measure the internal and external quality of fruit [23,24,25].

The main emphasis of this review is centered on the scholarly works that have been published within the last five years. Nevertheless, earlier seminal papers that have played an instrumental role in shaping the course of the respective methods and applications, as well as studies that were pioneering or representative of challenges and problems encountered in specific areas, are included as well in this review.

## 2. NIR Spectroscopy

### 2.1. Analytical Framework of NIR Spectroscopy

NIR spectroscopy is a non-destructive analytical technique that has gained widespread acceptance in various fields due to its ability to provide a qualitative and quantitative characterization of a broad range of samples [26,27,28,29,30,31,32,33]. In the applications reviewed here, particularly useful are sensors operating in the wavelengths belonging to the “conventional NIR” region, corresponding to wavelengths between 800–2500 nm (12,500–4000 cm^−1^). In particular, the spectra in conventional NIR can provide valuable information on the molecular composition, structure, and dynamics of samples without requiring any prior treatment or labeling. On the other hand, the Vis/NIR region usually refers to the range of wavelengths where the visible spectrum (400–700 nm) and the NIR region (700–2500 nm) overlap. Furthermore, often distinguished is also the SW-NIR region that refers to the short wavelength end of the NIR spectrum, typically between approximately 800–1100 nm (9000–12,500 cm^−1^); however, this division is rather arbitrary and depends on the source. In addition to these conventional definitions of the spectral regions, noted should be the increasingly popular instruments that operate over a broad wavelength window that extends over these commonly accepted boundaries. Mentioned here should be, e.g., portable multi-band spectrometers equipped with multiple detectors enabling them to acquire spectra over the entire Vis and NIR regions, and even include a narrow fragment of UV region, such as the ASD QualitySpec Trek Portable Spectrometer that operates over the 350–2500 nm window.

**Figure 2:**
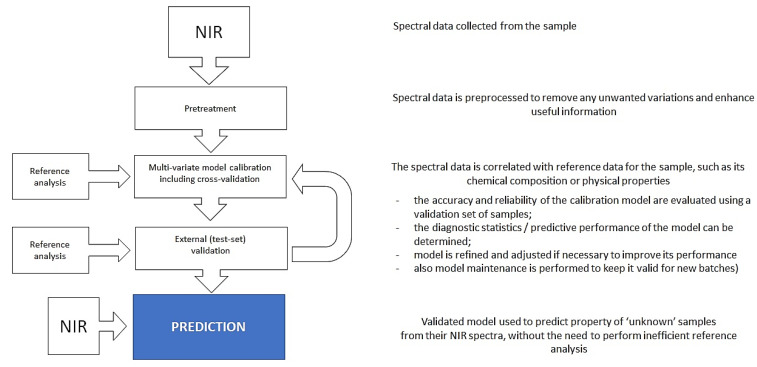
Scheme of the analytical framework in NIR spectroscopy on the example of quantitative analysis. A qualitative approach (i.e., classification) is analogous; however, reference data might be arbitrary in that case (e.g., “authentic” vs. “non-authentic” product; “bad” vs. “good” quality product, etc.). The ultimate outcome is the prediction of the property of the “unknown sample” (i.e., for which the inefficient reference analysis was deliberately skipped) towards which the model was calibrated.

This non-destructive method measures the absorption of light by molecules at specific wavelengths characteristic of their chemical bonds, which in itself is a quantitative process. However, for effective accessibility to this information in the analysis, a trained chemometric model is required to formally describe the relationship between the spectral intensity acquired from the sample and its property of interest, such as the concentration of a specific chemical constituent. Therefore, the analytical framework of NIR spectroscopy requires knowledgeable development of a robust data science method based on a properly calibrated, validated, and maintained model (Figure 2) [34,35]. Calibration (i.e., training) of a chemometric model involves measuring the spectra of a set of known samples and creating a calibration model that relates the spectral data to the sample’s chemical composition; in this process, reference (i.e., known) values associated with calibration and validation samples are needed. The calibration model is then used to predict the chemical composition of unknown samples based on their spectral data. Chemometric analysis can either be directed at the quantification of a selected compound or group of compounds of interest (e.g., proteins, fats, or moisture) or classifying samples based on their arbitrary property (e.g., authenticity, variety, or geographical origin), making it useful for quality control and process monitoring. NIR spectroscopy coupled with chemometric analysis can also analyze multiple components and perform multiple classifications from the sample spectral data simultaneously by using different calibration models.

Quantitative prediction of chemical constituents is achieved by fitting a multivariate regression model that describes the relationship between spectral data (i.e., multiple spectral points) and a set of reference values [34,35]. In principle, when the data set is sufficiently simple, this can be achieved even by a basic multiple linear regression (MLR), a simple linear method that relates the spectral data to the reference values using a matrix linear equation. Although fairly popular in the earlier applications of spectroscopy in the agri-food sector, often MLR and PCR can only be applied reliably when spectra are sampled at a few uncorrelated discrete points, which matched the capabilities of early spectrometers [36]. In more modern practice, high-resolution spectra often manifest strong collinearity (i.e., highly correlated two or more spectral variables carrying redundant information), and more advanced regression methods are recommended. Nowadays commonly used chemometric methods for solving the linear multi-variate regression problem include principal component regression (PCR) and partial least squares regression (PLSR); both approaches reduce the dimensionality of the spectral data by extracting new variables used to model the relationship between the spectral data and the reference values. This transformation can reduce the effects of collinearity on the regression coefficients. However, if collinearity is severe, PCR and PLSR can still suffer from instability or unreliable coefficients.

On the other hand, non-linear relationships between variables and samples can be described by, e.g., support vector machines (SVM), in which a hyperplane or set of hyperplanes is constructed in high-dimensional space; the SVM method can be used both for classification and regression. A separate family of non-linear methods is artificial neural networks (ANN) often used when the relationship between the spectral data is complex; however, these methods require extensive supervision and rich data sets for reliable use.

Classification methods aim to predict the class or category of a sample based on its spectral data [34,35]. One commonly used classification method is k-nearest neighbor (k-NN), which calculates the distance between a new sample and a set of training samples to assign it to the nearest class (i.e., category). Another widely used classification method is linear discriminant analysis (LDA), which finds a linear combination of spectral data that maximizes the separation between classes. Other classification methods include decision trees, random forests, and SVM. When applied to solve classification problems, SVM constructs a hyperplane or set of hyperplanes in a high-dimensional space, which can be used to separate sample classes. These methods can be enhanced through feature selection or extraction techniques to improve the accuracy of the classification model. Overall, the choice of classification method depends on the nature of the data, the number of classes, and the desired level of accuracy.

Among the most common set of data science tools applied in analytical NIR also PCA (principal component analysis), a dimensionality reduction method, should be mentioned. It is commonly used in exploratory data analysis to reduce the complexity of high-dimensional data sets by transforming the original variables into a smaller set of new variables, called principal components, which explain most of the variation in the data [34,35]. PCA is often used as a pre-processing step before applying other methods, such as regression or classification. On the other hand, clustering methods are also frequently employed in exploratory data analysis in analytical NIR, as they enable the grouping of samples based on similarities and the identification of potential outliers or clusters of interest [34,35].

Prior to solving the regression and classification problems in NIR spectroscopy, spectral pretreatment methods are commonly applied to reduce the detrimental effects present in experimental data sets that are not correlated with the property of interest of the sample (i.e., to improve the effective data quality), such as spectral noise or light scattering effects. These methods, including standard normal variate (SNV), multiplicative scatter correction (MSC), and derivatives, aim to preprocess the spectral data to improve the performance of the subsequent modeling methods. SNV and MSC are used to correct for variations in sample thickness and scattering effects, while derivatives can enhance the apparent resolution and highlight subtle features of the spectral data. The choice of spectral pretreatment method depends on the specific properties of the spectral data and the goal of the analysis [34,35].

A brief note should be given to the data fusion (i.e., sensor fusion, also in the literature referred to as multi-sensor fusion, MDF). It is the process of combining information from multiple sources to create a more complete and accurate picture of a system or phenomenon [37,38,39]. In the context of Vis/NIR spectroscopy, data fusion refers to the combination of spectral data obtained from different instruments or measurement techniques. The benefits of data fusion in Vis/NIR spectroscopy include improved accuracy and robustness of predictions, enhanced feature extraction, and increased flexibility in instrument selection and data acquisition. The recent literature demonstrates a growing interest in the application of this concept in the analysis of the vital properties of apples as well [40,41,42].

### 2.2. NIR Spectrometers and Miniaturization

The spectrometer typically consists of a light source, a sample holder, a monochromator or interferometer, and a detector [43]. The light source emits NIR radiation that passes through a monochromator or interferometer to isolate the specific wavelength range of interest. The radiation then interacts with the sample in the sample holder, and the transmitted or reflected radiation is detected by a detector. The most practical way of acquiring a NIR spectrum is by measuring the radiation diffusively reflected from the surface of the sample; contactless measurement is viable in such an approach. In some cases, the light source and detector can be integrated into a single device, such as a fiber optic probe. This probe can be inserted directly into a sample or used to collect reflected radiation from a sample surface.

**Figure 3:**
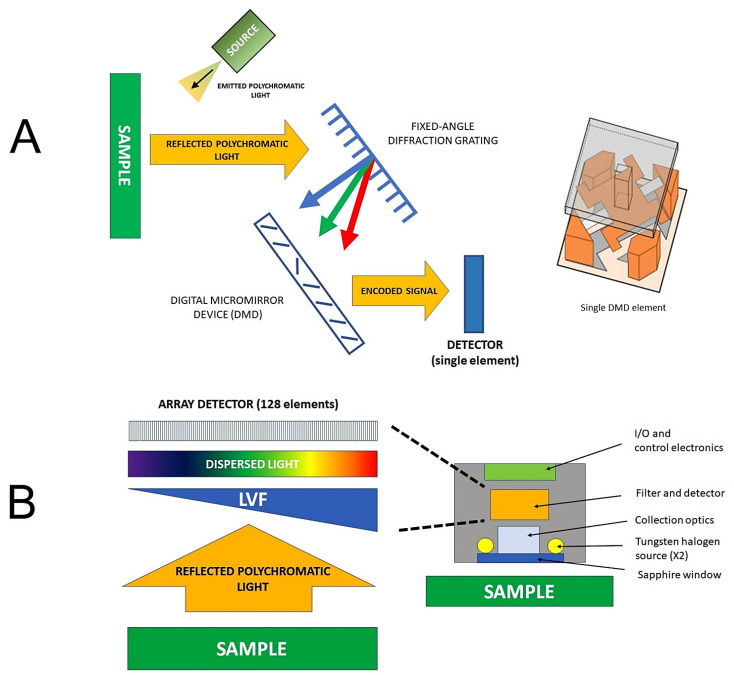
Examples of miniaturized NIR spectrometers; functional schemes of a design using digital micromirror device (DMD) (**A**) and a multi-channel spectrometer based on a linear variable filter (LVF) coupled with an array detector (**B**).

In recent years, novel technology led to the introduction of affordable miniaturized NIR spectrometers that can be easily operated by non-experts and offered as portable or handheld devices [44,45]. A number of competing engineering solutions exist in this area, for example, instruments based on a digital micromirror device (DMD) (Figure 3A). The DMD-based design uses a micro-electromechanical system (MEMS) device to select specific wavelengths and encode the signal that can be effectively measured by a single-element detector. On the other hand, a linear variable filter (LVF) coupled with an array detector, is a potent design of a multi-channel microspectrometer (Figure 3B). The LVF-based design uses a filter with a continuously varying transmission profile to isolate different wavelengths of light. Both designs are compact and offer good quality spectra, making them ideal for a wide range of on-site applications. This setup is used in the VIAVI MicroNIR 1700 ES spectrometer and enables collecting a high number of scans (i.e., integration time of 7.5 ns yielding spectrum in the region of 900–1600 nm; enabling the default measurement time consisting of 1000 averaged spectra in the total time of 7.5 s).

With the increasing miniaturization and portability of NIR spectrometers, it has become possible to perform analysis on-site, in the field, or even in remote locations, opening up new possibilities for applications such as environmental monitoring and agricultural analysis [46,47]. Designed to be lightweight, compact, and easy to use, making them ideal for field use, these instruments are becoming increasingly popular in many scenarios, particularly in the agri-food sector (Figure 3). Being suited for direct on-site operation, those instruments are particularly useful for food producers, processors, and quality control professionals who need to analyze samples in real-time to ensure product quality and safety. The examples of major step-ups in the applicability of NIR spectroscopy through handheld spectrometers include the measurement of the nutritional content of crops directly in the field, allowing for more accurate and timely decision-making regarding crop management and harvest [48]. On the other hand, these sensors can also be used to assess food quality during transportation and storage, reducing the risk of spoilage and waste.

### 2.3. Image-Based Methods

Briefly introduced should be image-based (i.e., spatially resolved) techniques are increasingly important in the reviewed area of applications, as they provide remarkable potential in delivering various essential quality parameters of fruits. Sketching the technical background of these diverse techniques extends beyond the capacity of this review, and the interested reader is pointed to focused monographies and articles devoted to these topics [49,50,51]. Here, only a brief summary of the relevant techniques will be provided to better expose the applications discussed in the following sections.

**Figure 4:**
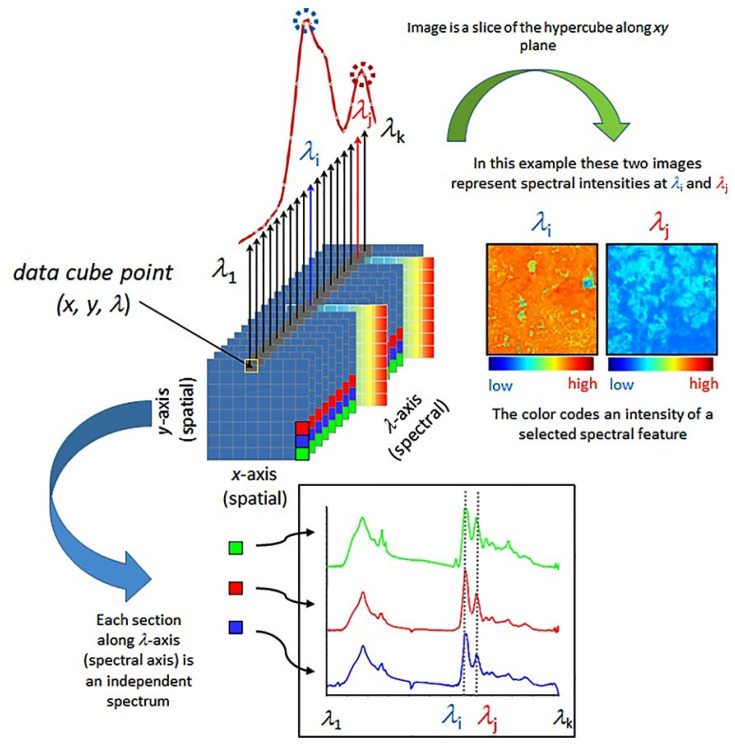
Simplified scheme presenting fundamental principles of a spectral data hypercube and visualization based on the most straightforward spectral information available, spectral intensity. Reprinted with permission from Ref. [56]. 2021, Elsevier.

Hyperspectral imaging and multispectral imaging are two spatially resolved techniques commonly used in apple analysis and quality control. Hyperspectral imaging involves capturing images at narrow wavelength intervals (i.e., at a relatively high spectral resolution; Figure 4). Multispectral imaging, on the other hand, captures images at a few selected wavelengths. Although not as detailed as hyperspectral imaging, multispectral imaging is faster and more cost-effective, making it a practical choice for industrial applications [49,50,51].

The spectral dimension of such images fundamentally contains the same information that is provided by point spectroscopy (Figure 4). This information can be incorporated into the analytical framework as described in Section 2.2, with an additional note that in this case also pixel-to-pixel or multi-pixel information in hyperspectral images is available for the analysis [52]. Hence, imaging in Vis and NIR regions is particularly suitable for the applications reviewed herein. These techniques can therefore provide information about the chemical composition of the sample, while also being suited to detect surface defects, bruises, and other forms of damage to the fruit. In addition, despite imaging instrumentation necessarily tending to be more complex than the practicality and advantages of the detectors, sources, and optics remain the same as for point spectrometers; factors which are also favorable in Vis/NIR wavelength window [53]. NIR hyperspectral imaging has been increasingly used in the food industry to study the chemical composition of various food products [54,55,56].

On the other hand, computer vision and machine vision are two related fields that involve the use of algorithms and software to analyze images and extract useful information. Most often this is performed for RGB (i.e., red–green–blue) images. Noteworthily, a four-band image RGB + NIR is often used, where the detector additionally captures a NIR band (i.e., most commonly, a single wavelength from the NIR region). The addition of the NIR band can provide additional information about the object being imaged, such as its moisture content or chemical composition (e.g., protein content which is not well manifested in RGB bands alone), which can be useful in quality control applications of fruits.

In the context of apple analysis and quality control, computer vision and machine vision techniques can be used to automatically classify and grade apples based on their appearance, size, and other physical characteristics. These methods can also be used to detect surface defects and other forms of damage to the fruit [57,58,59].

## 3. Analyzing the Quality Parameters of Apples; Internal and External Characteristics

The quality of apples can be assessed in terms of both external and internal characteristics [60] (Table 1). External quality refers to the physical appearance of the fruit, including factors such as color, size, shape, and surface defects [61]. Apples with bright and uniform color, free of blemishes, and desirable shape and size are considered to have high external quality, which can indicate freshness and potential for spoilage. In contrast, internal quality refers to the chemical and physical characteristics of the fruit’s flesh, such as taste (sweetness, sourness), texture (firmness, mealiness, crispiness, juiciness), aroma, and nutritional value (carbohydrates, proteins, vitamins) or internal defects (water core, frost damage, rotten). Factors that can affect internal quality include soluble solids content (SSC), sugar profile, total titratable acidity (TA), starch pattern index (SPI), phenolic compounds, and total dry matter concentration (DM). High internal quality apples typically have a balance of sweetness and acidity, a crisp texture, and a pleasant aroma [61,62].

To ensure that apples meet consumer expectations and are suitable for various applications, growers and producers must consider both external and internal quality parameters. For instance, fresh apples are expected to have high external quality and desirable internal characteristics, such as sweetness, acidity, and firmness. On the other hand, apples used for processing into juice, cider, or sauce may have lower external quality standards but must have high internal quality to maintain the desired taste, aroma, and texture. Therefore, a thorough evaluation of both external and internal quality parameters is critical for growers and producers to produce high-quality apples that meet different market demands [63].

### 3.1. Internal Quality Parameters

Internal quality parameters, e.g., taste, aroma, texture, and nutritional content are essential in determining the overall value of apples [61,62,64]. Factors that can impact internal quality, such as soluble solids content (SSC), total titratable acidity (TA), firmness, ripeness, starch pattern index (SPI), and total dry matter concentration (DM), have a direct effect on these sensory characteristics. The SSC, also known as the sugar content, typically measured as a percentage of the fruit’s weight, determines the sweetness of the fruit, which is a key factor in its taste. A higher SSC results in a sweeter taste, while a lower SSC leads to a tarter flavor. The TA, on the other hand, affects the fruit’s acidity, which is important for balancing out the sweetness and providing a pleasing taste. The natural acidity contributes to flavor and varies depending on the variety and growing conditions. The firmness of the flesh contributes to the texture and mouthfeel, with firmer flesh providing a crunchier texture. The ripeness and aroma of the fruit also play a role in its texture and freshness [61,62].

SPI measures the fruit’s starch content and can affect its texture, with a higher SPI indicating a firmer texture. Finally, DM concentration impacts the nutritional content of the fruit, with a higher concentration indicating a more nutrient-dense fruit. Proper growing conditions can ensure that the fruit develops its full flavor potential, while proper storage conditions can help preserve its sensory characteristics over time. Lastly, apples are a good source of nutrients such as vitamin C, fiber, and potassium, whose content varies based on the variety, growing conditions, and storage conditions.

Several factors can impact the internal quality of an apple, including genetics, environmental factors, and post-harvest handling practices [65,66,67]. Genetics plays a critical role in determining the natural sugar and acid content of the apple, as well as its texture and aroma. Environmental factors such as temperature, sunlight exposure, and soil quality can also affect the internal quality of the fruit by influencing the rate of sugar accumulation and other biochemical processes. Post-harvest handling practices, such as storage conditions and handling techniques, can also significantly impact the fruit’s internal quality. Proper handling practices are crucial to ensure that the fruit reaches consumers with optimal internal quality and flavor.

#### 3.1.1. Taste

Taste is one of the most important factors that influence consumer preferences [68,69]. Taste is a complex sensation that involves a combination of sweet, sour, bitter, salty, and umami (savory) flavors; in the case of apples, it results from various internal quality parameters, such as sugar content, acidity, and aroma, which can vary depending on the variety, growing conditions, and storage conditions [70,71,72,73,74]. The study of taste in apples is a crucial area of research for apple growers and processors. By understanding the factors that influence taste, they can develop strategies to optimize the internal quality parameters of the fruit, such as sugar content and acidity, to meet consumer preferences [68,75].

To better understand the taste of apples, several studies have been conducted using NIR spectroscopy, to correlate changes in the chemical composition of the fruit with taste attributes [76,77]. NIR spectroscopy has been used to measure the levels of various chemical compounds in apples, including sugars, organic acids, and volatile compounds, which can affect the fruit’s taste. Quantitative analysis of these compounds with NIR spectroscopy enables the prediction of the sweetness, acidity, and overall taste profile of the fruit [78,79,80,81]. For instance, Abu-Khalaf et al. [82] found that NIR can predict SSC and acidity, classify different varieties of apples based on taste characteristics with reasonable accuracy (>81%), and detect different varieties even when they had the same ratio of SSC and acidity. Instrumental methods such as penetrometry, double compression, and NIR spectroscopy, as well as sensory analysis, were used to predict the texture and taste of three apple cultivars [83].

#### 3.1.2. Aroma

Aroma is a quality parameter associated with the presence of volatile organic compounds (VOCs) in the fruit. Apples are known to contain over 350 volatile compounds [84], but only a small number of these have been identified as contributing to the fruit’s aroma [85]. The relevant VOC content, and the resulting aroma profile, can vary depending on the cultivar, ripeness stage, and storage conditions [85,86,87]. The majority of these volatiles are esters (78–92% of total volatiles), followed by alcohols (6–16%), aldehydes, ketones, and ethers, with varying amounts depending on the cultivar. Esters are the primary compounds responsible for the characteristic apple scent, and their concentration in both fresh and stored apples is determined by the number of ester precursors, such as lipids. These precursors are influenced by several factors, including the cultivar, growing conditions, harvest maturity, and storage conditions [88].

Aroma is an important quality parameter of apples that is closely related to taste. Apples with higher levels of SSC and lower levels of TA tend to have a sweeter aroma due to the presence of more volatile esters, while apples with higher levels of TA and lower levels of SSC tend to have a sourer aroma due to the presence of more volatile acids. The aroma of apples can change during storage, with some volatile compounds increasing in concentration and others decreasing. Overall, the aroma of an apple greatly influences consumer satisfaction and perceived freshness [89,90]. Therefore, monitoring changes in aroma during fruit development, maturity, and postharvest storage is important to maintain fruit quality and extend shelf life.

**Figure 5:**
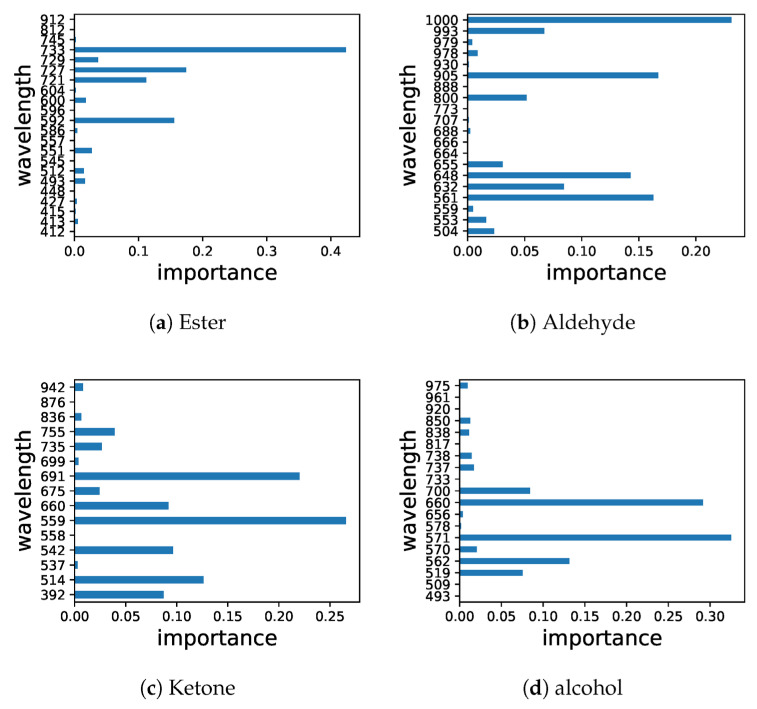
Distribution of characteristic spectra band ester (**a**), aldehyde (**b**), ketone (**c**), and alcohol (**d**). Reprinted with permission from Ref. [102]. 2022, MDPI (CC BY 4.0).

Conventional approaches include gas chromatography–mass spectrometry (GC-MS), solid-phase microextraction (SPME), and sensory evaluation are among the analytical techniques used to study apple aroma [91,92,93,94,95,96]. It should be noted that while NIR spectroscopy can provide useful information about the chemical composition of apples, it is not a perfect technique and may not be able to detect all aroma compounds or accurately predict the aroma characteristics of all apple varieties. Therefore, it is often used in conjunction with other analytical techniques and sensory evaluation to provide a more comprehensive understanding of apple aroma [83,97]. Nonetheless, NIR spectroscopy has been shown to predict the concentration of volatile compounds in apples that are responsible for the aroma [98]. The prediction models developed using NIR spectroscopy are reliable and accurate. The study by Ye et al. [99] showed that NIR spectroscopy is a useful method for determining volatile compounds in apple wines quickly and easily, without the need for costly and difficult chemical analysis. The results of this technique are comparable to those of the traditional GC-MS method and have the added benefits of being non-destructive and non-contaminating. However, spectroscopic methods may see competition from the GC in head-space mode (i.e., Flash GC) in this area of applications. The technique also offers a non-destructive and non-contaminating manner of analysis, while delivering a molecular fingerprint of volatiles by applying chemometrics to process the chromatograms; it has been successfully applied in the analysis of agri-food items including apples [100,101]. On the other hand, Zhu et al. [102] explored the use of hyperspectral imaging in a 400–1000 nm wavelength window (i.e., in Vis and Vis/NIR regions) for evaluating the aroma components of hybrid apple offspring. While the spectra-based model performed well in predicting chemical classes, it was less reliable in predicting individual chemicals. The study identified characteristic spectra for different chemical groups, with alcohol and ester being the most reliable (Figure 5).

#### 3.1.3. Texture

The texture is an essential quality attribute in apples well-exposed to the consumer. Texture analysis can provide information about the mechanical properties of apples, including hardness, chewiness, mealiness, and crispness [103,104,105]. The properties are mainly determined by cell wall structure and composition, which can change during the development, ripening, and storage processes of the fruit [106,107]. Various techniques have been used to study the texture of apples. Conventional mechanical testing and sensory analysis suffer from key limitations; the former being subjective to human judgments. The latter, while objectively assessing texture properties such as hardness and elasticity, may not fully reflect the sensory perception of texture. Imaging methods, such as X-ray and magnetic resonance imaging (MRI), deliver appreciable advantages here and can provide detailed information on the internal structure of apples and their texture; however, these techniques remain very costly, time-consuming, and limited to in-lab use [105,108,109,110,111,112,113,114,115]. In contrast, NIR sensors can be deployed on-site to perform highly efficient and rapid analysis of the chemical composition of apples, such as water, sugar, and starch content, which are closely related to texture. Several studies have shown that NIR can predict the texture of apples with high accuracy and the majority of the focus was directed at the assessment of firmness and mealiness, as discussed below in the Firmness section and Mealiness section. NIR spectroscopy can also predict the mechanical properties of apples, such as hardness, based on their chemical composition [116].

##### Firmness

Firmness (i.e., hardness) is a crucial indicator of the maturity, storability, and eating quality of apples. It is determined by the mechanical and structural properties of fruit tissue, and its measurement provides information about the texture, ripeness, and ability of the fruit to withstand handling and shipping [117]. It is affected by factors such as cultivar, storage conditions, and maturity level. The standard method for measuring apple firmness is the Magness–Taylor (MT) method, which involves the use of a penetrometer to measure the force required to penetrate the fruit flesh to a predetermined depth [118,119]. However, this method is time-consuming and requires a large number of samples to obtain accurate measurements [120]. The ripening process of the fruit is also an important parameter that affects its quality and shelf life [121].

Studies have shown that NIR spectra can be used to predict firmness values with high accuracy and the technique has the potential to be used for monitoring of apple firmness during processing and storage. The NIR spectra can be used to extract information about the chemical composition of the fruit, which is related to its firmness. Specifically, the ratio of absorbance values at specific wavelengths has been found to be strongly correlated with apple firmness [122,123,124,125,126,127].

Despite firmness not being directly expressed in spectral data, the suitability of NIR spectroscopy to deliver information that reliably correlate with this quality parameter of apples is evidenced in the literature. For instance, Vis/SW-NIR (460–1100 nm in this case) and hyperspectral scattering imaging (prototype online system) technique in the 450–1050 nm wavelength window were used to sort “Delicious”, “Golden Delicious” and “Jonagold” apples (sample set of *n* = 8491) into two quality grades based on firmness, SSC or the combination of both attributes [128]. In the classification performance of firmness, Vis/SW-NIR technique generally outperformed in that study. Good results in sorting with high consistency for firmness (ranging between 77.9% and 98.2%) and slightly less satisfactory sorting results for SSC (ranging between 62.0% and 91.7%) were obtained in that case.

A recent study by Mareckova et al. also confirmed the general suitability of NIR spectroscopy to deliver a useful analysis of firmness [129]. In that case, the relationship between the flesh firmness of apples and their chemical composition, specifically the content of water, pectins, and carbohydrates such as starch, was investigated. An excellent correlation was determined between flesh firmness measured using NIR spectroscopy and the classical invasive method, with accurate prediction values for varieties such as “Gala”, “Red Jonaprince” and “Jonagored”. It was concluded that the changes in firmness during storage were likely a result of variations in the analyzed components. However, the fruit quality was also found to be affected by a number of variables, including seasonal variations, tree age, the position of the fruit within the tree, light effects, location of the tree, and weather conditions. In a practical aspect, the method is suitable for use in automated commercial sorting lines, as it is non-destructive and highly cost-effective [129].

Osienko et al. [130] proposed a new method, based on a handheld Vis/NIR instrument for predicting the risk of disorder in Braeburn apples in relation to weather conditions and orchard management treatments. Noteworthily, the study involved a broad time window of three years for gathering data. Classification models were developed to assess internal browning, cavities, and fruit firmness after long-term controlled atmosphere storage. The results show a high success rate for predicting internal browning disorder and fruit firmness, with a 90% agreement between two separate years for internal browning disorder.

On the other hand, unorthodox approaches such as aquaphotomics that utilizes the water spectral pattern show potential as a valuable tool for investigating the texture sensory profiles of apple fruit. This is because water structures undergo changes in response to texture characteristics, regardless of the apple cultivar. Additionally, it is possible to differentiate apples with distinct texture sensory properties in a non-destructive manner. However, further research is necessary to fully comprehend the correlation between the water spectral pattern and pectin metabolism, as well as with the sensory profiles [131].

Noteworthily, NIR spectroscopy is readily suitable for designing cost-effective imaging instruments intended for the assessment of apple firmness, in which remote sampling is implemented via fiber probes. For example, a multifiber-based Vis/NIR spatially resolved (i.e., imaging) system was designed for simultaneous evaluation of SSC and firmness in apples [132]. The system uses 30 silica fibers connected to a Vis/NIR hyperspectral imaging camera to acquire spectral data and a light reference-free approach to calculate reflectance ratio spectra. The best-performing calibration models had coefficients of determination of approximately 0.97 for SSC and 0.96 for firmness, with root mean square errors of 0.20% and 0.37 Newtons (N), respectively. The method offers low-cost and portable detection of SSC and firmness for postharvest fruit evaluation.

##### Mealiness

Mealiness is a textural defect caused by the breakdown of cell walls in the fruit, which results in the release of excess juice and a loss of structural integrity. Several factors can contribute to the development of mealiness in apples, including cultivars, storage conditions, and postharvest treatments [133,134]. A better understanding of the factors that contribute to mealiness in apples could help growers and processors to minimize the incidence of this textural defect and improve the overall quality of apple products. To evaluate mealiness in apples, a range of techniques have been used, including sensory evaluation, ultrasonic, imaging, MRI, and NMR, as well as instrumental analysis of texture and water content [135,136,137,138,139,140,141,142]. The hyperspectral scattering technique is potentially useful for nondestructive detection of apple mealiness; however, improvements in classification accuracy are needed [143]. So far, the studies on the application of Vis/NIR spectroscopy show the potential of this technique when used in combination with other approaches. For example, Mehinagic et al. [144] analyzed the texture and taste of three different apple cultivars after prolonged storage using sensory and instrumental analysis including Vis/NIR spectroscopy. The aim was to predict the sensory perception of apple texture by instrumentally measured parameters. Penetrometry and compression were highly correlated with sensory textural attributes. A stepwise multilinear regression was performed on averaged penetrometry, compression, and Vis/NIR data for six sensory attributes. Spectroscopic data combined with physical parameters were successful in predicting complex sensations such as juiciness and mealiness in the mouth. At the same time, penetrometry was found to be more suitable for predicting sensory parameters corresponding to the quality of the fruit after harvest, while compression was more effective for predicting characteristics developed during storage.

#### 3.1.4. Nutrient Content

The nutrient content of apples has been the subject of many studies due to the fruit’s high nutritional value and health benefits [145,146]. Apples are a rich source of dietary fiber, vitamins, and minerals, including vitamin C, potassium, and various antioxidants. Numerous studies have investigated the nutrient content of apples using different analytical techniques, such as high-performance liquid chromatography (HPLC) and inductively coupled plasma (ICP) atomic emission spectroscopy [147,148,149,150,151]. These methods, however, can be time-consuming, labor-intensive, and may require sample preparation, making them less ideal for high-throughput analysis.

In contrast, NIR spectroscopy appears as a promising alternative for a rapid and non-destructive assessment of apple nutrient content. The technique has been found to be a reliable approach to quantifying the levels of nutritional compounds in apples [152,153]. For example, Pisaard et al. [153] investigated the use of NIR to determine the vitamin C and polyphenol content of apples in a breeding program focused on developing apples with high antioxidant content, scab tolerance, and high fruit quality. The study found that NIR could accurately determine sugar, acidity, and total polyphenol content, but the performance was less precise for maturity, firmness, and vitamin C content. The quality of the prediction and the determined ratio of prediction to deviation (RPD) values achieved in that study confirmed the suitability of NIR spectroscopy to perform the classification of the cultivars according to a range of concentrations. However, the potential for further improvement was deemed obvious for the quality of the prediction of vitamin C.

**Figure 6:**
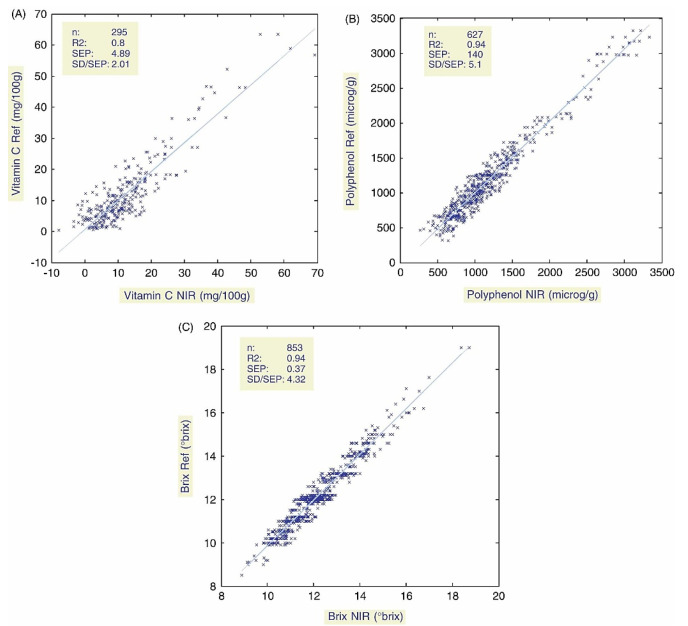
Prediction results for the validation sets. The figure shows the correlation between NIRS predicted and reference values for vitamin C (**A**), total polyphenol (**B**), and sugar (**C**) content obtained using the LS-SVM models. Reprinted with permission from Ref. [154]. 2013, John Wiley and Sons.

Accordingly, in another study, NIR with least squares support machine (LS-SVM) multivariate calibration was employed for the assessment of the quality of apples [154]. The models developed for vitamin C, total polyphenol, and sugar content showed good to very good prediction performance, with particularly high precision for sugar content. The low standard error prediction (SEP) values and relatively high RPD values indicate that NIR could allow for fine classification of apples according to their levels of vitamin C, total polyphenol, or sugar content (Figure 6). This could be especially useful in breeding programs where breeders are interested in classifying varieties according to a range of concentrations.

Successful determination of the properties of dried apple samples, including total phenolic matter, antioxidant activity, ascorbic acid, color characteristics, and spectral reflectance values was demonstrated by Cetin et al. [155]. These properties were determined from Vis/NIR spectra measured by a handheld instrument, alongside chromatic analysis, and biochemical properties as well. Different chemometric algorithms, such as ANN, k-nearest neighbor, random forest, Gaussian processes, and support vector regression, were used to estimate total phenolic matter, DPPH, FRAP, and ascorbic acid in that case.

Moreover, NIR spectroscopy has also been used to determine the mineral content of apples, including potassium and calcium. In this context, the bitter pit is a physiological disorder that affects apple fruit quality and causes significant postharvest losses, which are associated with a deficiency in minerals, mainly calcium. It is characterized by the appearance of brown spots on the fruit’s skin, which can lead to a bitter taste. Several studies have investigated the link between bitter pit and mineral nutrition in apple trees, with a particular focus on calcium. However, the mechanisms behind the disorder’s development and the factors that contribute to its severity remain unclear. Vis/NIR spectroscopy offers some potential in this area; however, studies so far point out certain limitations yet to be addressed.

Shoffe et al. [156] compared non-mineral and mineral-based methods for predicting bitter pit in susceptible apple cultivars, particularly “Honeycrisp”. Fruits were harvested three weeks before anticipated commercial harvest and at commercial harvest, and mineral contents in the peel were measured. In years 1 and 2, fruit were subjected to different treatments and stored at different temperatures, while in year 3 only the passive method was used. The passive and ethylene methods for fruit harvested three weeks before anticipated harvest showed higher or similar correlations with actual bitter pits after cold storage than mineral-based methods. The passive method was found to be more straightforward for commercial applications. In another study [157] Mogollon et al. demonstrated the feasibility of using NIR spectrometry for the early detection of bitter pit in “Fuji” apples, with accuracies between 60–70% for bitter pit (BP) severity <8 pits per fruit after only 10 days of storage, and between 80–90% for BP severity 8–9 pits per fruit. The models also show the possibility of detecting fruit prone to developing >8 pits during later storage. These results can help the apple industry to monitor the apple’s susceptibility to bitter pits soon after harvest, reducing postharvest losses and assuring quality for consumers. On the other hand, Kafle et al. [158] evaluated the use of NIR spectroscopy to detect bitter pits in Honeycrisp apples. Spectral reflectance data were collected from healthy and bitter pitted apples stored for different periods. Nine spectral bands were identified as associated with the bitter pit in Honeycrisp apples. The study found that both quadratic discriminant analysis (QDA) and SVM classifiers could be used to discriminate between healthy and bitter pitted apples with an average accuracy of 78–87%. These results suggest the feasibility of using NIR spectroscopy for detecting bitter pits in apples and further studies are underway to develop a portable device for apple bitter pit detection.

However, extracting information correlated with the elemental composition of the sample remains a challenging application of Vis/NIR spectroscopy. For instance, Bonomelli et al. [159] aimed to investigate the relationship between mineral composition and bitter pit symptoms in “Fuji” apples. Results showed that bitter pit-affected fruit had lower calcium concentrations and higher macronutrient ratios compared to healthy fruit. The study also found a correlation between the B/Ca ratio and BP incidence. However, the researchers concluded that it is not feasible to determine calcium concentration in apple fruit using Vis/NIR due to the lack of correlation observed between the reflectance spectrum and calcium concentration. Further research is needed to determine the role of boron in bitter pit disorder.

#### 3.1.5. Factors That Affect Internal Quality

##### Phenolic Compounds

Phenolic compounds are secondary metabolites widely found in plant tissues that play important roles in plant growth, development, and defense against environmental stresses [160]. Apples are known to contain a diverse range of phenolic compounds, including flavonoids, phenolic acids, and proanthocyanidins, which contribute to the fruit’s color, flavor, aroma, and nutritional value [161]. Phenolic compounds can significantly impact the taste, aroma, and texture of apples, with flavonoids contributing to bitterness and astringency, and procyanidins linked to astringency and hardness. Phenolic compounds in apples primarily exist in the peel and pulp and vary depending on cultivar, environmental factors, and post-harvest handling. Numerous studies have investigated the phenolic content and antioxidant capacity of different apple cultivars [162,163,164,165,166].

NIR spectroscopy has also been employed as a non-destructive and rapid method for determining the phenolic content of apples. For instance, a study by Pissard et al. [152] combined NIR with LS-SVM multivariate calibration to measure the phenolic content of apples. The models showed good to very good prediction performance, with particularly high precision. The low SEP values and relatively high RPD values suggested that NIR could allow for the accurate classification of apples based on their levels of total polyphenol content. Pissard et al. [167] also demonstrated the usefulness of NIR to measure the phenolic compounds and DM in apple peel and flesh separately, which could be helpful for improving fruit quality and storability. It was shown that the concentration of these compounds can vary greatly among apple cultivars and between the peel and pulp, making it difficult to predict their intake accurately. Specific apple varieties with a higher level of bioactive compounds in the flesh could be selected for breeding programs, and DM is an important characteristic related to fruit flavor and texture.

On the other hand, Beghi et al. [168] utilized a portable Vis/NIR system with PLSR to predict the total phenolic content in two apple varieties. The prediction accuracy differed between the two varieties, with better PLSR model parameters (*R*^2^ = 0.56; RMSECV = 0.06 mg catechin·g^−1^) obtained for “Stark Red Delicious” than “Golden Delicious” (*R*^2^ = 0.09; RMSECV = 0.10 mg catechin·g^−1^) due to the latter’s low phenolic concentration.

These exemplary studies highlight the potential of NIR spectroscopy as a tool for evaluating the phenolic content of apples, which can aid in the selection of cultivars with high levels of these beneficial compounds and in the development of healthier food products.

##### Soluble Solid Content (SSC)

The soluble solid content (SSC), also known as total soluble solids (TSS) content, is one of the most critical parameters to assess the quality of apple fruits. The sugars and acids, together with small amounts of dissolved vitamins, fructans, proteins, pigments, phenolics, and minerals, are commonly referred to as soluble solids and can be correlated with fruit properties such as sweetness, ripeness, maturity, and overall flavor [70,77]. While its higher value indicates a sweeter, and a lower indicates a sour apple, the SSC content can vary depending on various pre- and post-harvest factors, including the variety, climate, growing conditions, and storage time. The SSC in numerous fruits, consisting mostly (approximately 85%) of sugars (such as sucrose, glucose, and fructose) and sugar alcohols (for example, sorbitol and maltitol), is commonly determined by measuring the density or refractive index using a Brix scale hydrometer or refractometer. This value is expressed as “degrees Brix” (°Bx), which is equivalent to the weight percentage (%) of sucrose in a solution at a given temperature [169].

NIR spectroscopy has been widely used for the non-destructive analysis of SSC in apple fruit. It is an area of application, in which NIR spectroscopy has been particularly proven, with several studies reporting successful use of the technique in determining SSC content in apples [170,171,172,173,174,175,176,177,178,179,180]. Hence, attention will be given here only to the most recent research activity in this domain.

**Figure 7:**
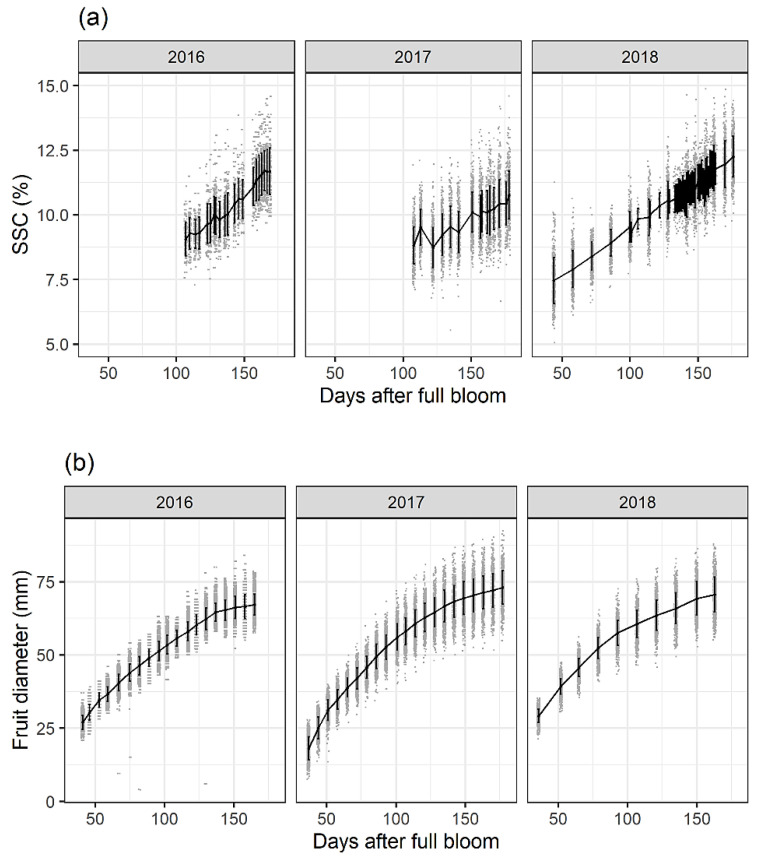
Soluble solids content (SSC) accumulation derived from the yearly calibrated partial least squares regression models (**a**) and fruit diameter growth (**b**) for the three study years and all treatments are shown. Mean values per measurement day are plotted as solid lines, single values as grey dots, and +/− standard deviations as black vertical bars. Reprinted with permission from Ref. [171]. 2021, MDPI (CC BY 4.0).

Recently, Biegert et al. [171] aimed to gain a better understanding of the non-destructive temporal development of SSC accumulation in apple fruit and to test the application of PLSR models of SSC in “Braeburn” variety with a focus on model transferability and accuracy. Noteworthily, a systematic investigation of the model performance over time was performed, which is an immensely meaningful factor in practice, e.g., for model maintenance. The multi-year model was found to be reasonable for overall performance, but yearly calibration models performed best for the same year (Figure 7). A sample size of 100 fruit for a yearly PLSR model with a wide range of SSC values is considered sufficient. The study also found that differences in sector position and crop load can lead to large differences in SSC during fruit development, offering the possibility for further physiological studies. In another study, Fan et al. [172] proposed a method to enhance the validity and robustness of the PLSR model over a long period and minimize the impact of biological variability on SSC prediction, making it suitable for practical applications. The study also investigated the long-term performance of a NIR calibration model for predicting SSC using apples collected from 2012 to 2018, taking into account the biological variability. These recent works well highlight the attention given to long-term model performance in practical scenarios of fruit quality monitoring.

On the other hand, the variability of fruit samples associated with different maturity stages and storage statuses can reduce the robustness of prediction models. Approaches such as local calibration were therefore investigated to improve the robustness by grouping similar samples and developing individual models to effectively handle the issue of batch-to-batch variation. Luo et al. [176] showed that the local calibration significantly improves the robustness compared to global calibration, especially when the prediction samples are of higher maturity. The authors suggested that the homogeneity of selected calibration samples, being of the same level of starch fractions, is the reason for the superior performance of local calibration. It was also reasoned that the modeling robustness can be further improved by including more samples from different regions and years.

Another issue related to the training set inhomogeneity was investigated by Tian et al. [177], who focused on the non-destructive determination of SSC of “Fuji” apple using an online spectra scanning system operating in Vis/NIR (615–1044 nm) region. The study proposed a method of “zone combination modelling” to evaluate the spectra acquisition geometry yielding, respectively, efficient and inefficient for SSC prediction. The results showed that the spectra of the apple core zone reduced the accuracy of SSC prediction and should be removed when developing prediction models. A selection of relevant wavelengths was also performed for the described purpose. Accordingly, an optimal prediction model was built using ten selected effective wavelengths, yielding root mean square error of prediction (RMSEP) of 0.733 and 0.61% for the prediction set, and a root mean square error of validation (RMSEV) of 0.721 and 0.71% for the validation set (SSC determination), respectively. The study concluded that the approach of zone combinations modeling is promising for the online detection of apple quality.

##### Sugar Profile

The predominant sugars found in apples are fructose, glucose, and sucrose, which contribute to the sweetness and flavor of the fruit. Various analytical methods have been employed to measure the sugar profile of apples, including HPLC and GC [74,181,182,183,184]. HPLC is considered the most accurate method for quantifying individual sugars, but it is time-consuming and requires expensive equipment while GC requires derivatization of the sugars, which can introduce errors. With the advent of NIR spectroscopy, there has been a growing interest in using this non-destructive technique for the rapid and accurate determination of sugar profile in apples. It can be used to measure constituent sugar concentrations in intact apples, with comparable performance to the reference HPLC method, but offers significant practical superiority [182].

Several studies have shown that NIR can predict individual sugars and total sugar content with high accuracy, making it a promising tool for the fruit industry [78,185,186,187,188,189]. This suitability of NIR spectroscopy was demonstrated relatively early. For example, Cho et al. [78] investigated the relationship between Brix and sugar content in apples using NIR spectroscopy. The correlation between Brix and total sugar content was found to be 0.66, while no clear relationship was identified between Brix and free sugar content. MLR analysis showed that the sweetness score, calculated with sucrose as a standard, could be determined by NIR spectroscopy with an R value of 0.8 and SEP of 0.82%. The study concluded that NIR spectroscopy could be used for the non-destructive evaluation of sweetness in apples. Following studies confirmed this, e.g., Temma et al. [186] demonstrated a correlation coefficient of 0.94 or more and a SEP of not greater than 0.546°Bx for four varieties of apples. For two types of apple juice, a SEP value of 0.439°Bx at most and correlation coefficients of 0.97 or more were obtained. That early study also identified 912 nm as an important wavelength for determining sugar content in both apples and juice.

The interest in this analysis continues in contemporary literature. For example, a design of an integrated NIR spectroscopy module for estimating sugar content in apples was recently presented by Byun [187]. The design process involved selecting effective wavelengths and reducing the analog-to-digital converter resolution. The final module used eight selected wavelengths and achieved a correlation coefficient of 0.365 and a standard error of calibration of 0.686 Brix. The module was implemented using a 0.18 μm 1P6M CMOS process and occupies a die area of 0.84 mm^2^.

**Figure 8:**
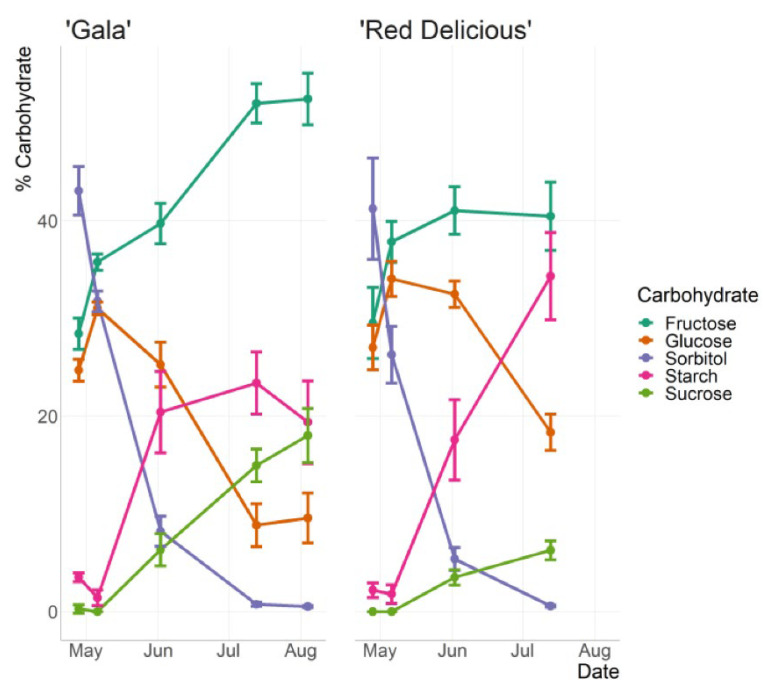
Percent contribution to total carbohydrates of fructose, glucose, sorbitol, starch, and sucrose from “Red Delicious” and “Gala” fruit collected throughout the 2020 growing season in Mills River, NC. Reprinted with permission from Ref. [189]. 2023, MDPI (CC BY 4.0).

On the other hand, a study by Larson et al. [189] aimed to monitor changes in the carbohydrate content of two apple cultivars throughout a growing season and evaluate the efficiency of the NIR technique to predict carbohydrates (Figure 8). The carbohydrate concentration in fruits during the growing season exhibited temporal patterns that are consistent with prior studies conducted on different cultivars. Sorbitol was identified as the primary carbohydrate in the early part of the season, while sucrose and fructose levels increased as the season progressed. Glucose and starch concentrations showed an increase until mid-season, after which glucose concentrations remained constant and starch concentrations decreased. The authors concluded that NIR spectroscopy is a suitable method for quantifying carbohydrates and predicting the levels of individual carbohydrates and total soluble sugars across cultivars, fruit cluster positions, and stages of fruit development during the growing season [189].

##### Total Titratable Acidity (TA)

Total titratable acidity (TA) is also an important parameter for the overall quality of apples, determining the flavor, taste, and freshness of fruits. Several studies have been conducted to determine the TA in apple fruit using various analytical approaches, including enzymatic and chromatographic methods [74,190,191]. Those techniques are highly sensitive and accurate but are destructive, require expensive equipment, and sample preparation, and are time-consuming. NIR spectroscopy offers a rapid and non-destructive alternative to traditional methods for the determination of TA in apple fruit. Highly accurate and reliable determination of TA in apples by NIR spectroscopy was demonstrated by numerous studies [192,193,194,195,196], with high correlation coefficients between the spectral data and the reference values obtained using traditional methods.

Refining the methods of TA analysis in apples by NIR spectroscopy remains an active direction of research. For instance, Pourdabani et al. recently investigated the wavelengths relevant to the prediction of pH and TA for Fuji apples [197]. Accordingly, the spectral band from 800 to 900 nm was selected for the analysis of the former parameter, and 830 to 910 nm for the latter one. The selection of these spectral bands was based on a trial-and-error approach, taking into consideration the peaks of interest in the spectra. The resulting model yielded a coefficient of determination (*R*^2^) of 0.86 for both parameters.

**Figure 9:**
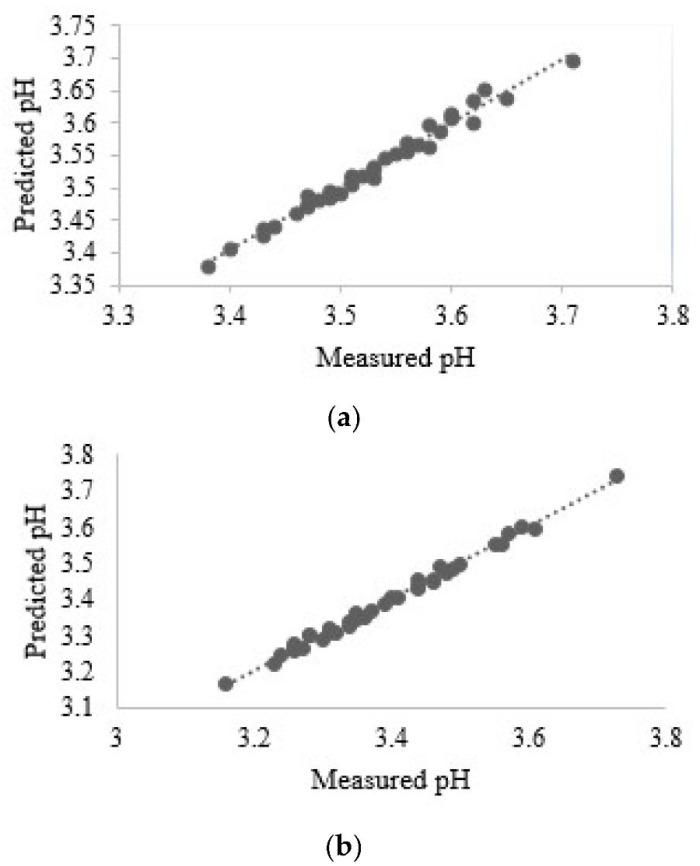
Predicted values of titratable acidity (TA) with the best developed models vs. its measured values for red apples (**a**) and yellow apples (**b**) in Vis/NIR spectroscopy. Reprinted with permission from Ref. [199]. 2022, MDPI (CC BY 4.0).

The study by Zhang et al. demonstrated the focus on refining the chemometric data analytical procedures for determining apple acidity using Vis/NIR spectroscopy [198]. In that work, the spectral data were pretreated with Savitzky–Golay (S–G) smoothing and wavelet transform. Subsequently, a modeling set was generated with successive projections algorithm (SPA) and a modeling candidate set with competitive adaptive re-weighted sampling (CARS). The optimal PLSR model, which reduced the number of selected wavelength variables from 129 to 36, achieved a determination coefficient of 0.9776 and a relative percent deviation of 6.6812. This study provided a promising reference for reinforcing the online determination of apple acidity. On the other hand, Hasanzadeh et al. examined various pre-processing procedures suiting the modeling of TA, alongside pH, SSC, and TP parameters using Vis/NIR spectroscopy applied to Red Delicious and Golden Delicious apples (Figure 9) [199].

##### Starch Pattern Index (SPI) or Starch Content Index (SCI)

Starch content is an important parameter in apple fruit quality assessment as it affects the texture and taste of the fruit during ripening and storage periods. The Starch Pattern Index (SPI) or Starch Content Index (SCI) is widely used to assess the starch content in apples as critical indicators of the fruit’s maturity and quality [200,201]. The SPI is a visual score of the microscopic appearance of the starch grains in the apple tissue, while the SCI is a quantitative measure of the starch content in apple tissue using chemical analysis. The determination of starch content in apples has traditionally been carried out by inefficient, destructive, and reagent-dependent enzymatic methods. This exposes the advantage of NIR spectroscopy as the spectral bands of starch deliver reliable information on the SPI and SCI parameters of apples. Several studies have demonstrated the effectiveness of this technique in this application, with high correlations between the NIR predictions and the reference values obtained by conventional methods [202,203,204,205].

Recently, Pourdabani et al. [206] used Vis/NIR spectroscopy to nondestructively predict tissue firmness, acidity, and starch content in Fuji apples at different ripening stages. An artificial neural network-cultural algorithm (ANN-CA) was used for non-linear regression, and the results showed that the proposed method was effective in estimating fruit properties. The mean coefficients of determination for firmness, acidity, and starch content were reported to be high when using only the three most effective wavelength spectral data.

On the other hand, NIR hyperspectral imaging has also been used to study starch content in apples, with promising results. Early demonstration of the feasibility of using NIR imaging spectroscopy as a tool for determining apple fruit maturity was established, circumventing the need for expert interpretation of traditional starch index assignments, which are subjective in nature [207]. In a recent study by Peirs et al. [208], hyperspectral images were collected from apple samples using a NIR imaging system, and the images were analyzed to determine the distribution of starch within the apple tissue. That study found that NIR hyperspectral imaging could accurately predict the starch content of apples, and the images could be used to create starch distribution maps within the fruit.

##### Total Dry Matter Concentration (DM)

Total dry matter concentration (DM) is another critical parameter that measures the amount of solid matter in the fruit as it affects the texture and flavor of the fruit [209]. DM is the sum of all solids in the apple fruit after drying and is calculated by subtracting the fruit’s moisture content from 100%. High DM concentration indicates a high proportion of dry matter in the fruit and is associated with better eating quality and longer shelf life. Conversely, low DM concentration indicates lower-quality fruit with a shorter shelf life [210,211,212].

**Figure 10:**
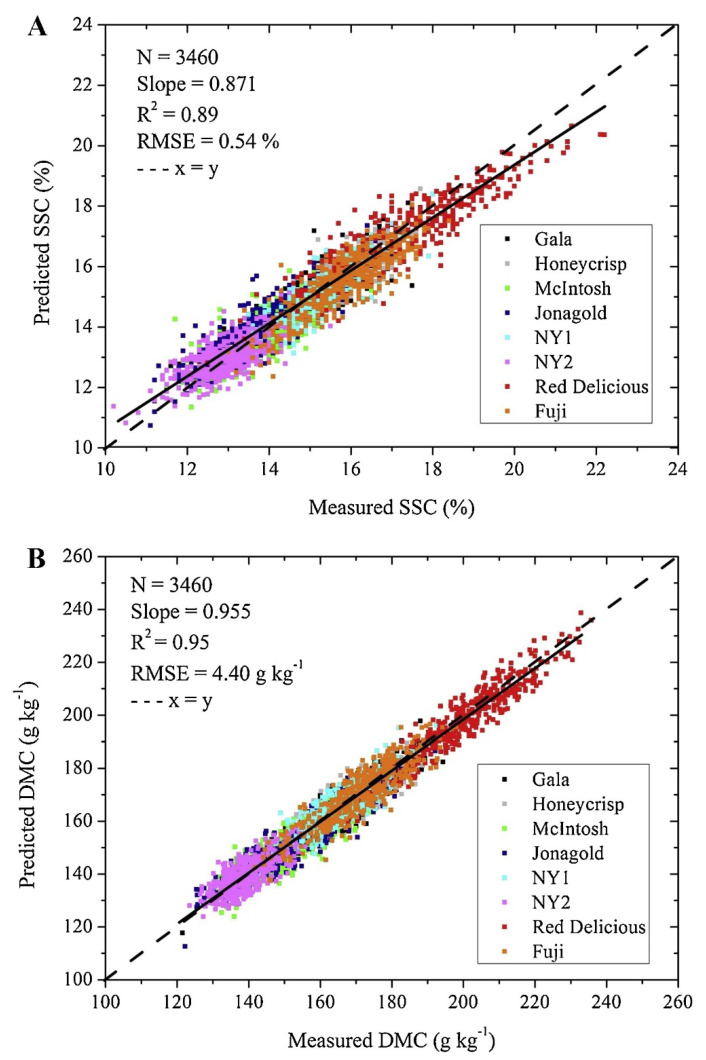
Scatterplots for external validation on (**A**) soluble solids content (SSC) and (**B**) dry matter content (DM) of multi-cultivar models. N = number of measurements; *R*^2^ = coefficient of determination of external validation; RMSE = root mean square error of external validation. The dashed lines represent the lines with slope = 1. Reprinted with permission from Ref. [216]. 2019, Elsevier.

NIR spectroscopy has been widely used to study DM concentration in apples. Several studies have reported the successful application of this technique to determine DM concentration in apple fruit, with high correlation coefficients between the predicted and reference values [167,213,214,215,216]. For instance, Travers et al. [215] evaluated the prediction performance of models for DM and SSC in apples over a storage period. While the RPD values for the models were below the minimum threshold required to consider the models strong enough for general quantitative predictions, they compared favorably to earlier achievements reported in the literature. On the other hand, Zhang et al. [216] developed NIR spectroscopy models to predict SSC and DM in several apple cultivars, including “Royal Gala”, “Golden Delicious”, “Elshof”, “Fuji”, and “Jonagold” (Figure 10). The study found that building individual cultivar models led to more accurate predictions, with “Jonagold” showing the highest *R*^2^ values. However, external validation revealed overfitting issues and data distribution challenges. To mitigate these issues, the study recommended using multi-cultivar models, which are more practical and robust for predicting SSC and DM in different origins, seasons, maturity stages, storage conditions, and periods. Recently, Zhang et al. [217] focused on the relationship between DM and SSC in “McIntosh”, “Red Delicious”, and “Fuji” apples. The study found that fruit DM and SSC at harvest were closely related, and the relationship was improved during maturation and storage. In this study, calibration models for predicting SSC and DM based on NIR spectroscopy were developed using PLSR. The models showed strong correlations with *R*^2^ values ranging from 0.77 to 0.85 for SSC and from 0.75 to 0.85 for DM. The RMSE of the calibration models was observed to be between 0.44 to 0.62% for SSC and from 4.25 to 4.92 g kg^−1^ for DM. The study demonstrated that NIR spectroscopy has the potential to be a non-destructive method for predicting SSC and DM in apples due to the strong linear relationship between the two parameters.

### 3.2. External Quality Parameters

External quality parameters of apples can be visually evaluated and assessed through touch. They include size, shape, color, and texture. Size refers to the overall dimensions of the fruit, such as its diameter and height, while shape describes its form, including its roundness or oblong shape. Color refers to the skin’s appearance, ranging from shades of green to yellow, red, or even dark purple. Texture refers to the physical characteristics of the skin, flesh, and core, such as firmness, juiciness, and crispness [61,62].

External quality parameters are critical in consumer selection, as they are the first characteristics noticed when choosing fruits for consumption [218,219]. Skin color is often used as an indicator of ripeness, with bright and uniform colors preferred over dull or spotted skin. Size and shape can also influence consumer preferences, with larger and more uniform fruits perceived as higher quality. Surface defects, such as bruises, cuts, or insect damage, can affect fruit quality and increase spoilage risk. These parameters play a significant role in consumer acceptance, as they determine overall quality and freshness. External quality parameters are also used for commercial grading and sorting, such as sorting by size or color to meet market standards or specific customer specifications, hence forming a critical property of fruits for both consumer acceptance and commercial purposes [220,221].

#### 3.2.1. Color

Color is a crucial quality parameter for apples that is used to determine the fruit’s maturity and ripeness [222,223,224]. The skin color of an apple is influenced by various factors, including genetics, environmental conditions, and postharvest handling [225,226,227]. The geographical locations of orchards can influence the color of apples as well [228].

Various techniques can be used to measure apple color, including visual assessment, colorimeter measurements, and image analysis [229,230,231,232]. While color is nominally manifested in the Vis region, its appearance results from the presence of chemical compounds that can be successfully analyzed in a broad spectral region, including Vis/NIR and conventional NIR as well. The vibrational bands of these chemical constituents are often more specific than broad electronic absorption features appearing in the Vis region. Anthocyanins and chlorophyll are the main pigments responsible for the color of apple skin [233,234,235]. Several studies have investigated color changes during fruit development, maturity, and postharvest storage [236]. The color of apple skin changes from green to yellow or red as the fruit ripens due to chlorophyll degradation and anthocyanin synthesis [237,238].

For example, Solovchenko et al. [239] demonstrated the use of Vis/NIR reflectance spectroscopy for estimating chlorophyll and carotenoid content and for estimating ripeness. The study estimated pigment content and on- and off-tree ripening rates and detected physiological disorders in apple fruit. Discussed were also the basic spectral features of fruit reflectance and their implications for method development. Merzlyak et al. [240] showed that Vis/NIR spectroscopy is a sensitive tool for determining the peel content of pigments in whole fruit during development. The assessment of peel pigments such as chlorophylls, carotenoids, and anthocyanins was demonstrated for five apple cultivars. Several reflectance indices were developed for estimating pigment content, which could be potentially useful for non-destructive assessment in the fruit of other plant species. However, further investigation is needed to improve the indices for other apple cultivars and achieve a more precise assessment.

**Figure 11:**
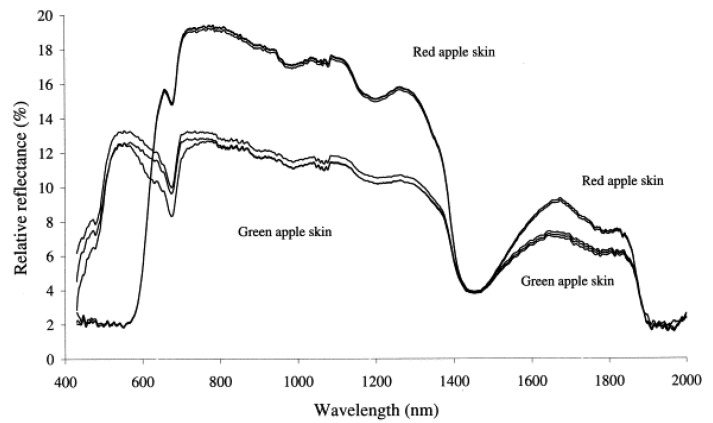
The skin reflectance (rskin) of green and red apple skin. Reprinted with permission from Ref. [241]. 2000, Elsevier.

Interestingly, the dependence of the penetration depth of electromagnetic radiation against the fruit tissue in different wavelength regions is a meaningful factor. It might be decisive for designing a specific application, depending, e.g., whether the property of interest of the apple is manifested in superficial parts of the fruit or its deeper parts. For example, the study by Lammertyn et al. [241] compared two optical configurations in the analytical method developed for predicting sugar content in apples using NIR spectroscopy. The study also developed a technique to measure light penetration depth in apple tissue, with values ranging from 2 to 4 mm depending on the wavelength (Figure 11). In this regard, the property of the sample itself, such as its color, can be seen to be meaningful as well. The study suggested further research on the light penetration properties of apple tissue in the 1300–2500 nm range and other apple cultivars.

More recently, Ye et al. [242] explored the potential of a UV/Vis/NIR interactance device in determining the degree of red coloration in the flesh of a red-fleshed apple variety “Kurenai no Yume”. The results indicated a significant correlation between the interactance spectra and the anthocyanin content in the apple flesh, suggesting that a non-destructive and fast interactance technique can be developed for estimating the degree of red coloration in red-fleshed apples.

On the other hand, recent attention is also directed at further improving the feasibility of these techniques in a practical sense. Abbaspour-Gilandeh et al. reported a cost-effective approach to the estimation of the chlorophyll *b* content of red delicious apples using color and NIR spectral data combined with hybrid ANN [243]. The authors concluded that the cost of the Vis/NIR spectroscopy system setup is important for real-time applications, and a small window of around 680 nm wavelength could be used to reduce the expenditure of the analysis. The spectral method outperforms the color method in terms of determination and regression coefficients and error estimation parameters. Additionally, it was demonstrated that using effective spectra selected by the hybrid ANN-differential evolution algorithm as input to a hybrid ANN biogeography-based algorithm improves the results in that case.

#### 3.2.2. Size

Apple size is a significant factor in the apple industry, as it influences not only marketability and consumer acceptability but also production efficiency [244]. Therefore, continuous attention is directed at this quality parameter [245,246,247,248,249,250,251,252]. Apples are typically classified into different size categories based on their diameter; mass and aspect ratio of fruits can be used to classify normal and misshapen apples [253].

While influenced by growing conditions and nutrient availability, apple size is mainly determined by genetic factors [254,255,256,257,258]. Apart from marketability and consumer preference, apple size also correlates with internal quality parameters [61,62]. Thus, NIR spectra of intact apples can provide information on the size and internal structure of the fruit based on variations in absorption patterns caused by different components such as starch or SSC. Several studies have demonstrated that NIR spectroscopy can accurately predict apple size and size distribution with high precision. Larger apples tend to have higher SSC and lower acidity levels than smaller ones. In addition, larger apples may have lower firmness than smaller ones due to their higher water content.

In recent years, NIR spectroscopy has been repeatedly used to predict apple size and size distribution. Jiang et al. [259] investigated the impact of apple size on its spectrum and the prediction performance of the PLSR model of apple SSC. It was found that apple size differences can affect the spectrum, and the relationship between apple size and its spectrum light intensity satisfies the logarithmic function. Different solution methods and preprocessing models were studied to address the poor performance of the SSC prediction model due to apple size differences. The authors concluded that the inclusion of fruit diameter as a variable in the size compensation model for SSC enhances the prediction performance of the model and satisfies the need for online detection of SSC in apples with varying fruit diameters.

The same authors proposed a method to correct the NIR spectra of apples with varying sizes to improve the accuracy of the SSC prediction models [260]. The method involved standardizing the transmission spectra using extinction coefficients and correcting them based on average values. After size correction, the spectra were modeled to predict SSC, resulting in a significantly improved correlation coefficient and RMSEP of PLSR. The proposed method reduces the influence of fruit size variation on SSC models, making it suitable for improving the performance of NIR online inspection devices for apples of different sizes.

**Figure 12:**
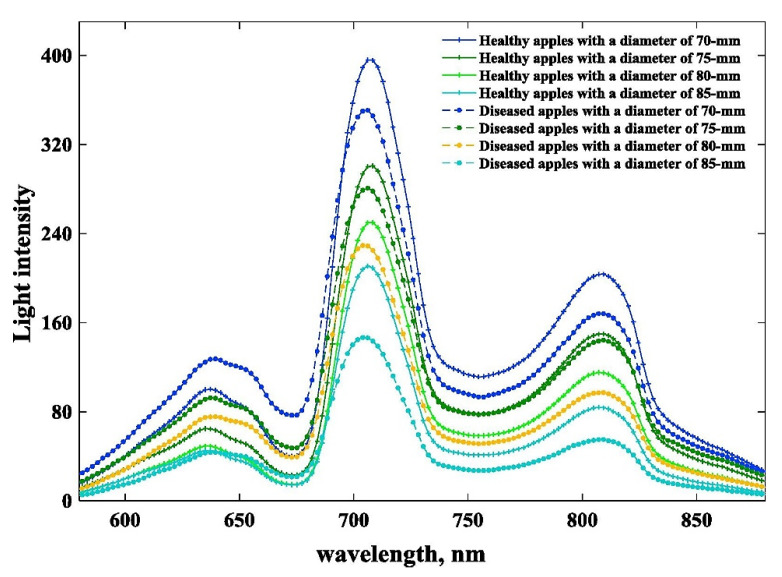
Comparison of spectral information for different sized healthy and diseased apples. Reprinted with permission from Ref. [262]. 2019, Elsevier.

The relationships between the size of the fruit and its optical properties attract attention in this context. Vaudelle et al. [261] used Monte Carlo simulations to investigate the impact of apple size and skin on the measurement of optical properties of the flesh using steady-state reflectance measurements and diffusion approximation. The study found that the skin layer had little influence on the retrieved internal optical parameters of an apple, except for measurements close to the source. On the other hand, Tian et al. [262] proposed a new method to correct transmission spectra based on fruit size for identifying diseased fruit. To standardize the transmission spectra of apples with varying sizes, the study obtained the extinction coefficient of transmitted light. The extinction coefficient was then used to correct the transmission spectra of apples based on average fruit size. Classification models were developed using the corrected spectra (Figure 12). The accuracy of the models using corrected spectra was superior in identifying healthy apples with large transverse diameters and diseased apples with small transverse diameters compared to the models using original spectra.

#### 3.2.3. Shape

In addition to size, the shape of apples is another important factor that affects their marketability and consumer preferences [263]. It is determined by a complex interplay of genetic, environmental, and physiological factors [264,265]. The shape of the apple is also related to its internal quality, with some studies indicating that more elongated apples tend to have higher SSC and less acidity [264]. Therefore, the shape of the apple is an important characteristic to consider when evaluating apple quality and determining its intended use [264].

Image-based methods are naturally best suited for the assessment of this parameter, and these approaches garnered significant interest for that purpose from the outset. For instance, Cheng et al. [266] proposed a dual-camera approach using NIR and MIR for online detection of apple stem-end/calyx to avoid incorrect sorting, achieving a 100% recognition rate for good apples and 92% recognition rate for defective apples, indicating potential for reliable online sorting of apples for defects. On the other hand, it was identified that conventional 2-D machine vision techniques (i.e., a two-dimensional image that represents the fruit’s surface from one angle only; no depth information included) for apple sorting and grading, often face challenges in distinguishing apple stem-end/calyx from defects. Zhu et al. [267] proposed an interesting 3-D-based approach to overcome this hindrance, utilizing data processing methods that reconstructed the 3-D surface of apples from 2-D NIR images using the shape-from-shading method, achieving an overall detection rate above 90%.

**Figure 13:**
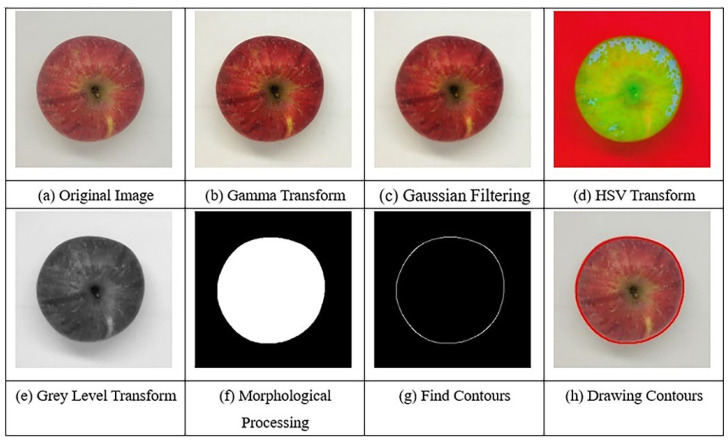
Image preprocessing process. (**a**) Original image (**b**) gamma transform (**c**) Gaussian filtering (**d**) HSV transform (**e**) grey level transform (**f**) morphological processing (**g**) find contours (**h**) drawing contours. Reprinted with permission from Ref. [268]. 2022, Public Library of Science (CC BY 4.0).

Recently, Wang et al. [268] focused on analyzing the principles of apple defects, shape, size, and Brix detection and grading based on China’s national standard. The study established appearance quality classifier models based on machine vision (Figure 13) and constructed a Brix value prediction model based on NIR spectroscopy. The results showed high accuracy in apple grading detection, and the Brix prediction model had the best performance with the CARS-PLS model. The study suggests that improvements in algorithms and equipment can further enhance the accuracy of the system and that it can be applied to other round fruits and vegetables.

#### 3.2.4. Surface Defects

Surface defects in apples, such as bruises, scabs, or insect damage, can greatly impact the quality and market value of the fruit. Image-based techniques, such as computer vision, hyperspectral imaging, fluorescence imaging, and NIR imaging are highly potent techniques for the assessment of apple quality in that respect [269,270,271,272,273,274,275]. These non-destructive methods can provide detailed information on the size, shape, and location of the defects and accurately classify those as well [276,277].

Applications of these techniques have been a focus of attention for this purpose since early on. For example, Bennedsen et al. [271] developed a machine vision system for sorting apples based on NIR images and characterized its analytical figure-of-merit in this role. The system used visible grey-scale images to verify orientation and images acquired through optical filters for defect detection. Defects were detected using different segmentation routines and an ANN-based routine. The system performed well in detecting individual defects and measuring their area, with a range of 77–91% and 78–92.7%, respectively, across eight apple varieties.

**Figure 14:**
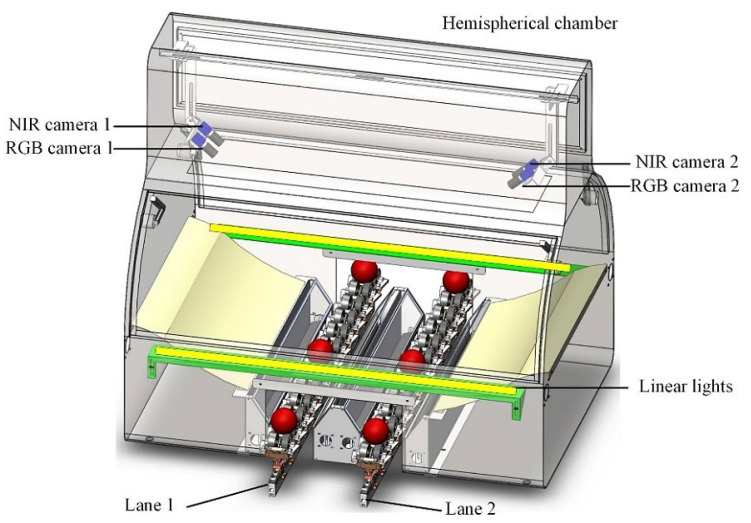
Inspection module for two-lane fruit sorting machine. Reprinted with permission from Ref. [278]. 2022, Elsevier.

Despite the numerous studies that have been conducted in this area, there are still ongoing efforts to improve the accuracy and reliability of apple sorting and grading systems. Recently, Fan et al. [278] presented an online apple defect detection method using combined RGB and NIR cameras, and a diffuse illumination chamber (Figure 14). The data analytical procedure was based on a deep learning algorithm tailored for improved detection speed. The proposed method achieved a 93.9% detection accuracy on different cultivars of apples at the online test assessing five fruit per second and showed potential to be implemented in commercial packaging lines for the identification of fruit defects.

## 4. Miscellaneous

### 4.1. Identification/Origin/Authenticity

**Figure 15:**
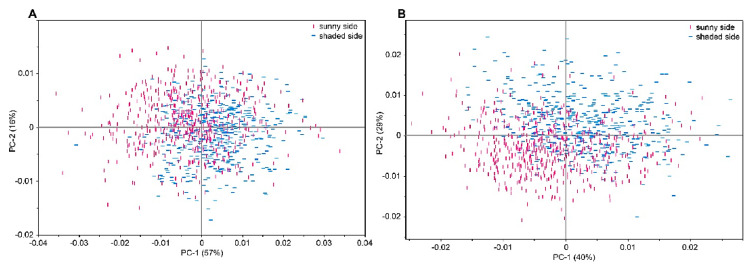
PCA plot of all apple NIR spectral data acquired from the sunny and shaded side of the apples (**A**) 511 “Braeburn” apples on both sunny and shaded sides (**B**) 539 “Cripps Pink” for the sunny side and 533 apples for the shaded side. Reprinted with permission from Ref. [282]. 2015, MDPI (CC BY 4.0).

The traceability of food products is of great importance in the food industry, particularly in the fruit supply chain. In the context of apple traceability, NIR combined with chemometrics has been successfully used to classify apples based on their origin, cultivar, and quality. It is possible to develop predictive models for the classification of apples by Vis/NIR spectroscopy according to their origin and cultivar, forming a highly practical and flexible method for the traceability of apples [279,280,281]. For example, Eisenstecken et al. demonstrated that high accuracy rates in classifying apples according to their cultivar and orchard elevation can be delivered by spectroscopic methods in the conventional NIR region [279]. The technology could be applied in the fruit supply chain, for instance in warehouses to control the origin of apples at delivery or as a test method for the recently introduced EU “mountain product” label. Eisenstecken et al. [282] also explored the potential of NIR spectroscopy as a tool for post-harvest management of fruit quality. That study found that NIR can identify different cultivars and freshly picked vs. stored fruit, as well as the sun-exposed side of apples with increased nutrient content (Figure 15). It was also concluded that combining NIR spectroscopy with other analytical techniques can lead to more efficient, reagent-free tools for post-harvest management.

While the performance of this technique in such application is well-established nowadays, various chemometric approaches were investigated in combination with NIR spectroscopy for further refinement of the method [283,284,285,286,287]. Recently, Xu et al. [288] proposed a similarity-based particle swarm optimization combined with the PFCM algorithm (SPSO-PFCM) to quickly and accurately distinguish apple varieties using NIR diffuse reflectance spectra. The algorithm maintained particle diversity and avoided premature convergence. Compared with other fuzzy algorithms, SPSO-PFCM had better classification performance and achieved accuracies of 96.66% and 93.33% for the meat and IRIS data sets, respectively. The study concludes that NIR diffuse reflectance combined with the SPSO-PFCM clustering is an effective method for classifying apple varieties.

NIR has repeatedly been proven to identify apple cultivars and growing regions [289,290,291,292]. For instance, He et al. [290] successfully applied NIR spectroscopy on apple skins to distinguish “Fuji”, “Red Delicious” and “Royal Gala”; Bobelyn et al. [291] investigated “Golden Delicious” apples from four different countries by NIR and Wang et al. [292] focused on “Fuji” apple from 7 different apple production sites in China. On the other hand, Tian et al. [293] studied how the FT-NIR spectral analysis of SSC values in apples was affected by geographical region variability. Single-region models performed well when test and training samples were from the same region but poorly when predicting SSC values from other regions. To reduce the effect of region variability, two multi-region prediction models were proposed and compared.

Improvements in the performance of such analysis can be delivered by optimization of the sampling. For instance, Schmutzler et al. [294] found that using surface scanning with NIR improved the accuracy of determining the geographical origin of apples. An automated surface scanning technique was compared to the commonly used measurement technique, and the results showed that automated non-destructive surface scanning led to a more robust analysis. Multivariate clustering was performed, and successful PCA was realized to identify Golden Delicious apples from different regions using the spectroscopic data set derived from surface scanning. The method also included quantitative predictions of SSC, total acid, and polyphenol content in Golden Delicious and Pink Lady apples and delivered a reduction in the prediction errors.

### 4.2. Evaluation of Apple Maturity

Evaluation of apple maturity is a crucial aspect of the fruit industry as it determines the appropriate time for harvesting and post-harvest management [295,296,297]. It can be evaluated based on physical, chemical, and sensory properties [298,299,300,301,302]. From the point of view of the spectroscopic techniques, the general property is therefore closely connected to the analytical problems described in the previous sections of this review. Several studies have reported the use of NIR spectroscopy to evaluate apple maturity based on various parameters such as sugar content, acidity, and firmness [22,303,304,305]. The practicality of NIR spectroscopy makes it an ideal method in this role, enabling optimizations of postharvest management and overall enhancement of quality control in the apple industry [306]. Hyperspectral imaging in the NIR region was repeatedly used to estimate apple quality and maturity with considerable success as well [22,304,305,306,307,308,309].

### 4.3. Optimization of Storage Conditions

Storage time is a crucial parameter that affects the quality of apple fruit, including its texture, flavor, and overall quality [310,311,312,313]. Apples can be stored for extended periods without losing their quality, but the length of storage time is dependent on a number of factors, such as the cultivar, temperature, humidity, and atmospheric conditions [314,315,316]. Vis/NIR spectroscopy has been widely used to evaluate the stored apples and to monitor changes in fruit quality during storage [317]. These changes in the chemical and physical properties of apples during storage, such as loss of firmness, changes in sugar and acid content, and development of off-flavors and discoloration, can be monitored with these techniques. Particularly useful is the ability to detect internal defects, such as watercore and decay, which can develop during storage but may not be visible on the surface of the fruit and are challenging to detect online during production [318,319,320,321,322].

Several studies have reported the use of NIR spectroscopy to predict the storage time of apples based on changes in their chemical and physical properties [323,324,325,326,327,328]. For instance, Liu et al. [324] proposed a method for rapid determination of Fuji apple storage time using Vis/NIR spectroscopy combined with PCA. The results showed that PCA alone could not discriminate apples with different storage times using original spectroscopy data, but it successfully discriminated them when signal-to-noise ratio (SNR) maximal values were analyzed as well. On, the other hand, Zhang et al. [325] examined the quality changes in Fuji apples at three different maturity levels during cold storage. Results showed that the fruit quality deteriorated with prolonged storage, with a decrease in firmness and an increase in SSC; PCA clustering suggested that starch content may be the underlying factor. Ignat et al. [326] used Vis/NIR and SW-NIR spectrophotometers to measure the SSC, titratable acidity, and firmness of apples at harvest and after 2, 4, and 6 months of 0 °C storage. The best *R*^2^ values were for SSC and starch, while titratable acidity and firmness predictions were less precise. Camps et al. [329] evaluated the ability of Vis/NIR spectroscopy to classify apples left on the shelf or stored in a cooled room. The classification of storage modalities was analyzed using factorial discriminant analysis (FDA) for three apple cultivars. The results showed that Vis/NIR spectroscopy allowed for the correct classification of fruits of each cultivar by more than 95%, and the classification of storage modalities was over 75% and 83% for fruits stored in a cooled room and shelf, respectively.

**Figure 16:**
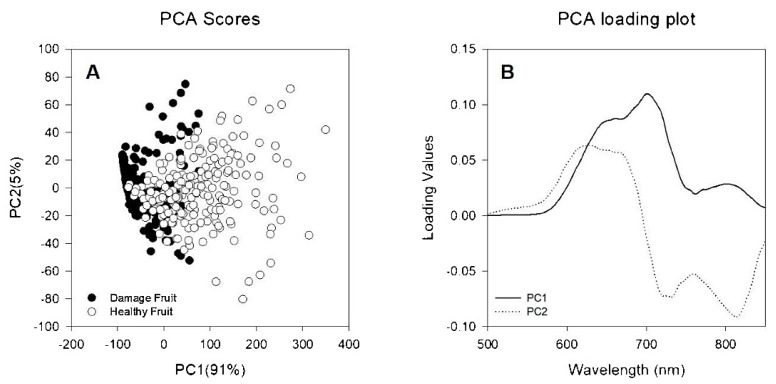
Principal component (PC) analysis of “Cripps Pink” apple semi-transmittance spectra between 600 and 830 nm after 150 d plus 7 d at 20 °C. (**A**) Score plot. Black dots correspond to damaged fruit, white dots correspond to healthy fruit. (**B**) Loading plot. Continuous line shows loading values for PC1, dotted line shows loading values for PC2. Reprinted with permission from Ref. [333]. 2020, Elsevier.

On the other hand, internal browning is a type of tissue breakdown that occurs in the flesh of the apple, often without any visible external symptoms. Thus, the appearance of this defect in stored fruits also poses a challenge for quality monitoring procedures. Several studies have investigated the use of NIR or Vis/NIR spectroscopy for detecting and quantifying internal browning in apples [330,331,332,333]. For example, the study of Mogollon et al. [333] suggested that quantitative models based on semi-transmittance acquisitions between 100–1100 nm can accurately predict the severity of internal browning tissue in “Cripps Pink” apples after 150 days of storage as early as 90 days of storage (Figure 16).

Noteworthily, attention is given to increasing the universality of the models taking into account varying conditions of the measurement. For instance, Guo et al. recently compared the effects of temperature on the models and used preprocessing methods and variable selection methods to improve the prediction performance under varying conditions [334]. The results showed that the NIR system could accurately predict the quality attributes of apples stored for different periods, with promising results for firmness, SSC, titratable acidity, and vitamin C.

### 4.4. Quality Compromise Effects Related to Storage Conditions

Scald and cold scald are two common physiological disorders that affect the quality of apples during storage at low temperatures [335]. Scald appears as dark, greasy spots on the fruit’s skin, while cold scald is a white, waxy discoloration. Despite only resulting in superficial damage to the fruit, these disorders compromise the shelf value of apples causing significant economic losses for apple growers and processors. Cold scald is particularly problematic as it is inflicted by cold storage, which is otherwise highly important for preserving the freshness of apples for prolonged storage time. Understanding the mechanisms behind these disorders and developing effective strategies to manage them is critical. Factors such as storage temperature, duration, fruit maturity, and the presence of ethylene can all influence the development of scald and cold scald in apples. Antioxidants and modified atmosphere storage are among the physical and chemical treatments that have been investigated for their ability to prevent or reduce the incidence of these disorders [336,337,338,339].

Reliable assessment of apples towards the presence of chilling injury by NIR spectroscopic sensors is a promising measure for minimizing the loss during storage. In particular, early detection of the disease before it leads to visible changes in fruit skin condition will offer a decisive advantage. A joint Austrian–Italian consortium was created for the comprehensive dissection of the superficial scald in apples [340,341]. The fundamental objective of this project was to uncover innovative regulatory mechanisms of superficial scald in apples, offering a fresh set of resources valuable for the scientific and technical communities involved in apple breeding and postharvest.

Zanella et al. [342] investigated the potential of NIR to trace the scald susceptibility of apple fruit. PCA was applied to NIR spectra of Granny Smith apples with different scald susceptibility generated by different temperature conditioning treatments before storage. The results showed that NIR is a promising non-destructive technology to discriminate apples based on their scald incidence during storage and reflected the effect of scald mitigating storage preconditioning treatments. The goal is to develop this approach into a postharvest decision support system to minimize the loss of horticultural products.

## 5. Use of Portable/Handheld NIR Spectrometers

In recent years, the use of portable or handheld NIR spectrometers has become increasingly popular for the analysis of food products [44,45,343]. These instruments offer a convenient and cost-effective alternative to traditional benchtop spectrometers, as they can be used in the field or on the production line for rapid, non-destructive analysis. The key advantage of portable/handheld NIR instruments is their ability to provide direct on-site analysis outside the laboratory while preserving all other practical advantages of NIR spectroscopy, which is particularly useful in agri-food applications [46].

### 5.1. Current Analytical Potential of Miniaturized Spectrometers in Apple Quality Analysis

Miniaturized NIR or Vis/NIR instruments were extensively applied in the analysis of various properties of apples [46,47,344,345,346,347,348,349,350]. Among those applications, the prediction of the SSC parameter in apples has been particularly frequently studied using those instruments [170,171,216,351,352,353,354,355,356]. Therefore, attention will be given here to the most recent accomplishments in this topic.

For instance, Zhang et al. [355] used a handheld SSC analyzer for internal quality detection of apples based on NIR spectroscopy. The data handling/analysis followed an increasingly popular cloud service and a smartphone application for user control. The prediction models based on the extreme learning machine (ELM) method were reinforced by data pretreatment and optimization with several algorithms including MSC, SNV, S-G smoothing, linear weight reduction of extreme learning machine combined with the improved particle swarm optimization (IPSO-ELM). The resulting model achieved an *R*^2^ of 0.993, RMSEP of 0.0155, and RPD of 11.6, outperforming traditional approaches, and NIR spectroscopy was found to be a reliable nondestructive method for SSC measurement in apples.

On the other hand, Ying et al. [344] aimed to develop a fast and non-destructive method for detecting apple sugar content using a portable NIR spectrometer. The sample set was divided using Kennard–Stone (K–S) algorithm, and the optimal wavelength range for sugar detection was determined using PLSR and interval PLSR (i.e., iPLSR). The best prediction model for apple sugar content was established using the PLSR method, with an optimal range of 1198–670 cm^−1^ after comparing nine pretreatment combinations. The model with first-order differentiation, S-G smoothing, and standard normalization showed the best performance with a correlation coefficient of 0.9223 and RMSEC of 0.423 for the correction set, and a correlation coefficient of 0.9189 and RMSEP of 0.237 for the prediction set. A portable spectrometer with low accuracy could be used for fast and lossless detection of apple sugar.

**Figure 17:**
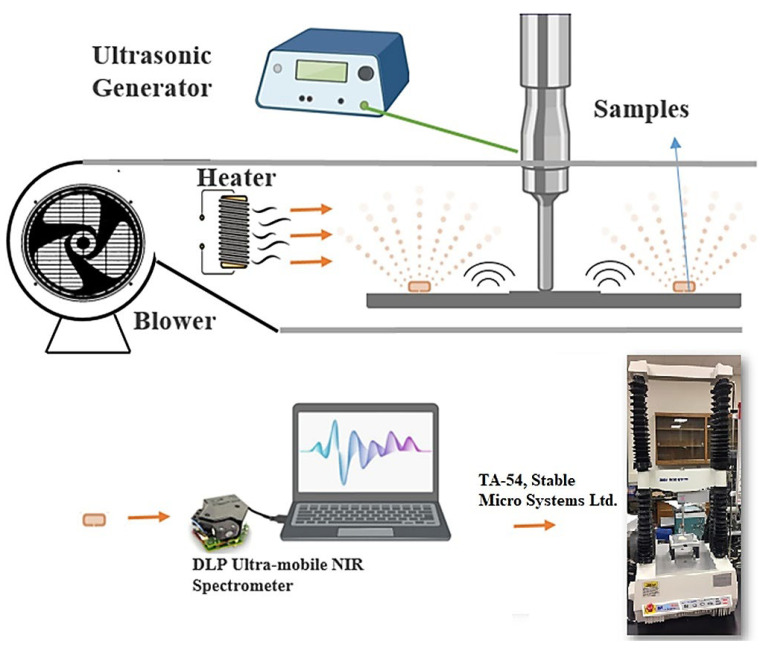
Schematic of ultrasonic contact dryer. Reprinted with permission from Ref. [116]. 2022, Elsevier.

Interestingly, recently, Malvandi et al. [116] reported a novel, non-destructive and cost-effective method for measuring apple hardness during ultrasonic contact drying using a portable NIR spectrometer (Figure 17). Linear PLSR and MLR algorithms were used for multivariate analysis of NIR spectra to develop a calibration model for hardness. In addition, partial least squares–artificial neural networks (PLS–ANN), a machine learning algorithm that combines the strengths of PLSR and ANN methods, with the former being used to reduce the dimensionality of the data set, while the latter models the non-linear relationships between the input and output variables. In the reviewed study, the nonlinear PLS–ANN hybrid method was found to significantly improve the correlation between absorbance spectra and hardness. The best PLS–ANN calibration model achieved a correlation coefficient of 0.95 and an RMSEP of 11.49 N and only seven feature wavelengths were needed for successful prediction. As a next step, Malvandi et al. [357] used a portable NIR instrument for non-destructive and real-time assessment of moisture content in apple slices during direct contact ultrasonic drying. PLSR and Gaussian process regression (GPR) models were used to develop the calibration model for predicting moisture content and drying curve. In the best case, accurate prediction with *R*^2^_p_ = 0.99 and RMSEP = 3.32% was yielded, while only three wavelengths were included in the model. The thin-layer drying model was developed by the equation that was in the best agreement with the reference measurements.

### 5.2. Assessment of Analytical Performance and Specific Applicability

Miniaturized sensors have varying designs and multiple competing engineering solutions exist in this area, factors which introduce differences in their applicability and performance profiles in different analytical scenarios [44,45]. This topic attracts considerable attention also in the context of apple analysis, and several studies have been conducted to compare the performance and accuracy of portable NIR devices and their applicability potential in various setups. The significance of such studies was recognized early; for instance, a comparison of three NIR spectrometers including two portable units was performed in 2009 by Paz et al. [358]. The study evaluated several NIR instruments in terms of accuracy for the measurement of soluble solid content, firmness, and shelf-life of apples.

Recently, Kaur et al. [359] compared the performance of different portable NIR instruments with a benchtop instrument for predicting DM of three different main fruit types (apples, kiwifruit, and summerfruit). The stationary instrument showed the best performance with high prediction *R*^2^ values, while the hand-held instruments delivered moderate to high *R*^2^ values. However, caution was recommended when evaluating the relative performance of different instrument types or formats based on data generated with just a single instrument or data set. A comparison of the performance of benchtop and selected handheld spectrometers for determining apple quality parameters was also conducted by Pissard et al. [360]. MicroNIR spectrometer offered performance equivalent to that of the benchtop spectrometer. In the second part of their study, different regression methods were used to develop calibration models for apple quality parameters using a large historical database acquired using the benchtop spectrometer. The LS-SVM method presented better predictive performance, and calibration transfer between the benchtop and handheld spectrometers was successful using the direct standardization method.

**Figure 18:**
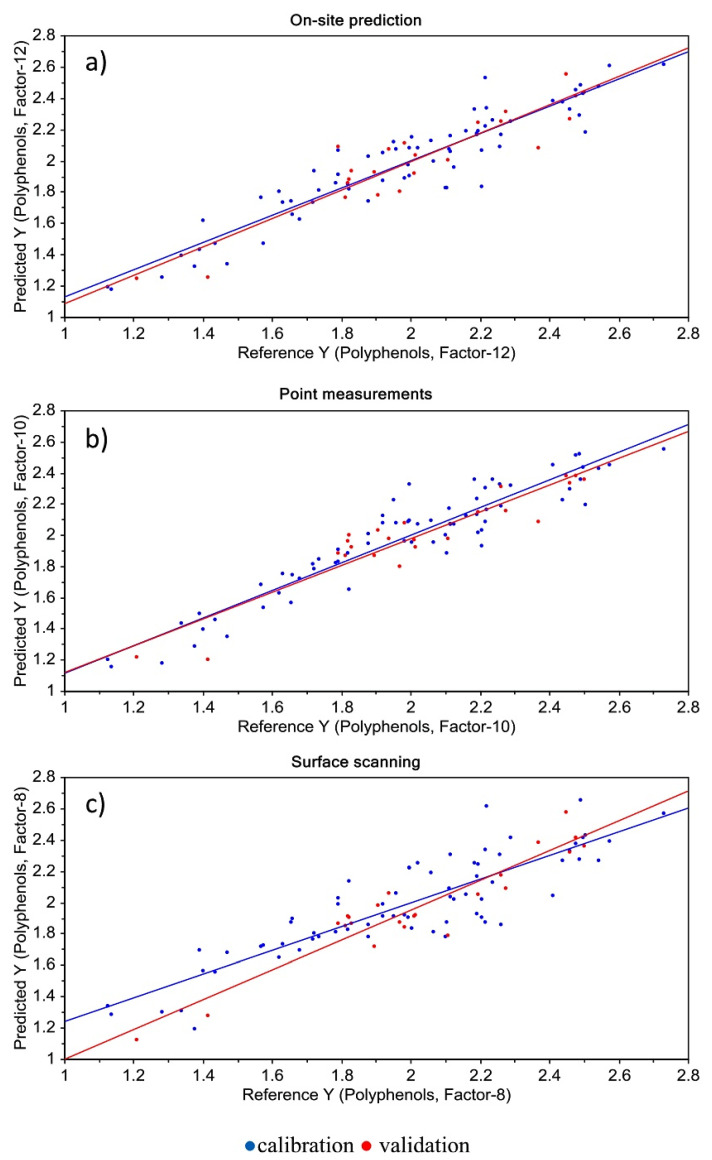
Prediction of the polyphenol content with three different instruments and methods: (**a**) on-site analysis, (**b**) point measurements, and (**c**) surface scanning. Prediction vs. reference values with regressions for calibration and validation. Reprinted with permission from Ref. [361]. 2016, Elsevier.

A similar aim was presented in the study by Schmutzler et al. [361] who examined the potential of a portable NIR spectrometer for non-destructive on-site analysis of apple quality attributes. The results demonstrated that the instrument was able to predict the total sugar content and concentration of polyphenolic compounds in the peel of apples of different varieties with satisfying accuracy (Figure 18). The portable device was characterized by portability, easy handling, flexibility, and the possibility to analyze apples directly on the tree without the need for sample preparation. However, the precision of high-end benchtop instruments was not reached in that case.

### 5.3. Calibration Transfer

In the last few years, the problem of calibration transfer (i.e., domain adaptation) attracted much attention in applied spectroscopy. Firstly, this procedure enables adjusting the prediction model of a spectrometer to maintain accuracy in different conditions. Secondly, transferring a calibration model from a benchtop spectrometer to a miniaturized one can reinforce the practicality of the analysis, improving even further the efficiency and flexibility of the on-site, real-time measurements [362]. To address this issue, different calibration transfer methods have been developed and studied to improve the transferability of calibration models between spectrometers [362]. Furthermore, in the case of agricultural applications, profound variance due to different factors such as batches (i.e., batch-to-batch variance), orchards (i.e., locations), and other complex effects originating, e.g., in season, conditions or temperature can affect the spectral profiles of apples and can impact the performance of models. In such cases, the model transfer is a relevant technique that can be used to at least partially mitigate these issues.

Recently, Li et al. [354] aimed to predict the SSC in apples using portable devices and to compare different calibration transfer methods for improving the accuracy of prediction. Results showed that the master and slave devices could predict SSC effectively, but direct use of the calibration model from the master device to the slave device was not practical. PDS (piecewise direct standardization) was found to be the most effective transfer method for spectra transformation, and optimization of the window size and the number of standard samples was necessary for satisfactory performance. The slope/bias (S/B) parameter was used to reduce prediction deviations between different batches of apples, and the proposed method effectively compensated for spectral response differences between the portable devices and different apple batches.

On the other hand, Li et al. [363] developed a strategy using wavelength selection and the transfer learning algorithm to discriminate the origin of Fuji apples between two NIR portable spectrophotometers. The study found that the combination of the manifold embedded distribution alignment (MEDA) method with a relative error analysis (REA) step to select wavelength (i.e., MEDA-REA) has yielded the model with the highest classification accuracy (Table 2). That combined strategy provided a low-cost approach to decrease the complexity of the modeling process. The study suggested that more waveband screening methods for calibration transfer should be explored to further reinforce the practicality of calibration transfer in routine applications by simplifying the transference model.

## 6. Summary

Spectroscopy in Vis/NIR and NIR regions has gained remarkable popularity as a non-destructive analytical tool for the quality assessment of apples. It enables rapid and non-invasive measurement of multiple quality attributes, including firmness, sugar content, acidity, nutrient content, and phenolic compounds. Spectroscopic techniques offer significant benefits, such as time-saving, eliminated/reduced sample preparation and analysis costs, and the ability to perform rapid measurements in real time. The non-destructive nature of the application of Vis/NIR and NIR sensors allows for repeated measurements on the same sample without altering its integrity for increased robustness, while multiple quality parameters can be assessed simultaneously from every single scan.

Numerous studies have demonstrated the effectiveness of Vis/NIR and NIR techniques in this area of application. This technology accurately predicts multiple quality parameters of apples and has great potential in the food industry. The remarkably rich literature on this topic reflects a continuous interest in developing new methods and refining the existing approaches in the field of quality assessment of apples, reflecting the importance of this field of application in the contemporary agri-food sector.

Although spectroscopic techniques in Vis/NIR and NIR regions have shown significant potential for non-destructive quality assessment of apples, there are still several open challenges and future prospects in this field. Due to the high diversity of fruit samples, it is important to address the challenge standing before the measurement procedure itself as well as the difficulties posed by the data analysis. Emphasized here should be the need to standardize the measurement protocols and refine the existing approaches to achieve robust calibration models that can account for the variations in apple cultivars, maturities, and environmental factors. Another challenge is the development of cost-effective and portable spectroscopic devices that can be more easily integrated into fruit packaging lines for rapid and real-time quality assessment, making these technologies more widely applicable. Overall, the continued research and development of spectroscopic techniques for apple quality assessment hold promise for enhancing the efficiency, quality, and safety of the apple supply chain.

## Figures and Tables

**Table 1:** General overview and categorization of the quality parameters of apples commonly analyzed in practical scenarios.

Quality Parameters	
Internal	External
Color	Taste
Shape	Texture
Size	Aroma
Surface defect	Nutritional value
	Internal defect

**Table 2:** Prediction results of these models. Bold figures represent the best results. Reprinted with permission from Ref. [363] 2022, MDPI (CC BY 4.0).

Calibration Transfer	Spectral Variables	Accuracy	Fuji-1		Fuji-2		Fuji-3		Fuji-4	
			Recall	Precision	Recall	Precision	Recall	Precision	Recall	Precision
TCA-REA	68	0.8739	0.9189	0.8095	0.9302	0.8333	0.5581	0.8571	0.9744	0.9744
BDA-REA	81	0.9160	0.9459	0.9091	0.9535	0.8039	06744	1	1	0.9630
**MEDA-REA**	**77**	**0.9454**	**0.9324**	**0.9583**	**0.9302**	**0.8889**	**0.9070**	**0.8864**	**0.9872**	**1**
